# FOXS1 is regulated by GLI1 and miR-125a-5p and promotes cell proliferation and EMT in gastric cancer

**DOI:** 10.1038/s41598-019-41717-w

**Published:** 2019-03-27

**Authors:** Sen Wang, Longke Ran, Wanfeng Zhang, Xue Leng, Kexin Wang, Geli Liu, Jing Song, Yujing Wang, Xianqin Zhang, Yitao Wang, Lian Zhang, Yan Ma, Kun Liu, Haiyu Li, Wei Zhang, Guijun Qin, Fangzhou Song

**Affiliations:** 1 Molecular Medicine and Cancer Research Center, Chongqing Medical University, Chongqing, 400016 China; 2 Department of Biochemistry and Molecular Biology, Chongqing Medical University, Chongqing, 400016 China; 3 Department of Bioinformatics, Chongqing Medical University, Chongqing, 400016 China; 4 Department of Radiology, Affiliated Hospital of Southwest Medical University, Sichuan Province, 646000 China; 5 Information Technology Office of Chongqing Medical University, Chongqing, 400016 China; 6 Chongqing Public Health Medical Center, Chongqing Medical University, Chongqing, 400016 China; 7 Department of Gastrointestinal Surgery, The First Affiliated Hospital of Chongqing Medical University, Chongqing, China; 8 Department of Endocrinology of the Frist Affiliated Hospital of Zhengzhou University, Zhengzhou, Henan 450052 China

## Abstract

Gastric cancer (GC) is the fourth most common malignant neoplasm and the second leading cause of cancer death. Identification of key molecular signaling pathways involved in gastric carcinogenesis and progression facilitates early GC diagnosis and the development of targeted therapies for advanced GC patients. Emerging evidence has revealed a close correlation between forkhead box (FOX) proteins and cancer development. However, the prognostic significance of forkhead box S1 (FOXS1) in patients with GC and the function of FOXS1 in GC progression remain undefined. In this study, we found that upregulation of FOXS1 was frequently detected in GC tissues and strongly correlated with an aggressive phenotype and poor prognosis. Functional assays confirmed that FOXS1 knockdown suppressed cell proliferation and colony numbers, with induction of cell arrest in the G0/G1 phase of the cell cycle, whereas forced expression of FOXS1 had the opposite effect. Additionally, forced expression of FOXS1 accelerated tumor growth *in vivo* and increased cell migration and invasion through promoting epithelial–mesenchymal transition (EMT) both *in vitro and in vivo*. Mechanistically, the core promoter region of FOXS1 was identified at nucleotides −660~ +1, and NFKB1 indirectly bind the motif on FOXS1 promoters and inhibit FOXS1 expression. Gene set enrichment analysis revealed that the FOXS1 gene was most abundantly enriched in the hedgehog signaling pathway and that GLI1 expression was significantly correlated with FOXS1 expression in GC. GLI1 directly bound to the promoter motif of FOXS1 and significantly decreased FOXS1 expression. Finally, we found that miR-125a-5p repressed FOXS1 expression at the translational level by binding to the 3′ untranslated region (UTR) of FOXS1. Together, these results suggest that FOXS1 can promote GC development and could be exploited as a diagnostic and prognostic biomarker for GC.

## Introduction

Gastric cancer (GC) is the fourth most common malignant neoplasm and the second leading cause of cancer death. With approximately one million diagnosed cases and over 700,000 deaths recorded annually, GC is the third most common cause of cancer deaths worldwide^1^, and is frequently undiagnosed until a relatively advanced stage. The current optimal approach for GC therapy is surgical resection with curative intent and adjuvant chemotherapy or radiotherapy. However, the recurrence rate of GC remains high^2^ and clinical responses to other inhibitors for GC at the genetic level have been disappointing^3^. Because of the high molecular heterogeneity in GC, additional diagnostic and prognostic biomarkers and therapeutic targets are needed for improving clinical outcomes.

Fox proteins are a family of evolutionarily conserved transcriptional regulators defined by a common DNA-binding domain (DBD) termed the forkhead box or winged helix domain, and involved in a wide spectrum of biological processes, such as metabolism, development, differentiation, proliferation, apoptosis, migration, invasion and longevity^4,5^. Furthermore, FOX genes are differentially expressed in a large number of cancers; their role can be either oncogenic or tumor suppressive, depending on the family member and cell type^6^. FOXO3a modulates WNT/β-catenin signaling and suppresses EMT in prostate cancer cells^7^, while FOXM1 promotes pancreatic cancer EMT and metastasis via upregulating the expression of the urokinase plasminogen activator system^8^. The FOXM1 transcription factor network is activated in over 84% of cases of high-grade serous ovarian cancers via involvement in the homologous recombination DNA damage and repair pathway^9^, and FoxC2 has been linked to tumorigenesis and the progression of colorectal cancers through the Akt/GSK-3β/Snail pathway^10^, while FOXF2 suppresses gastric cancer through a novel FOXF2-IRF2BPL-β-catenin signaling axis^11^. Currently, FOXS1 is known to be expressed only in the sensory nervous system, and the role and involved mechanism of FOXS1 in gastric cancers have been rarely reported.

The Hedgehog (Hh) signaling pathway is crucial for growth and is often associated with human cancers^12^. Glioma-associated oncogene 1 (Gli1), a key transcription factor and a terminal effector of the HH cascade, has been shown to affect EMT in GC cancer cell lines and to influence lymphatic metastasis^13^. Although GLI1 elicits signal amplification by regulating target genes, the number of confirmed targets of the GLI1 transcription factor, for example, Patched (PTCH1 and PTCH2) and Human Hedge-hog-Interacting Protein (HHIP), is surprisingly small^14^. Yumei Diao *et al*. recently reported that FOXS1 was one of the top targets of the GLI1 gene and was shown to act in a negative feedback loop limiting the cellular effects of GLI1 in medulloblastoma and rhabdomyosarcoma cells^15^. However, the interplay of GLI1 and FOXS1 in GC cells has not been reported.

MicroRNAs (miRNAs) are a class of small 22-nucleotide-long endogenous noncoding RNAs that regulate the expression of genes at the posttranscriptional level by interacting with their 3′UTRs in a sequence-specific manner^16^, resulting in mRNA degradation or translational inhibition^17^. miRNAs are abnormally expressed in various cancers, and deregulated miRNA expression is strongly associated with tumor initiation, promotion and progression^18^. Therefore, it is urgently necessary to acquire knowledge regarding whether regulate the FOXS1 gene in GC and which miRNAs are involved.

Here, we determined the overexpression of FOXS1 in GC. FOXS1 identified as a target gene of GLI1 was most abundantly enriched in the hedgehog signal pathway. In addition, miR-125a-5p could bind the 3′UTR of FOXS1 and regulate the expression of FOXS1 via translational repression in gastric cancer cells, which may lead to further advancements in the knowledge of gastric cancer tumorigenesis.

## Results

### FOXS1 is highly expressed in gastric cancer

**Figure 1:**
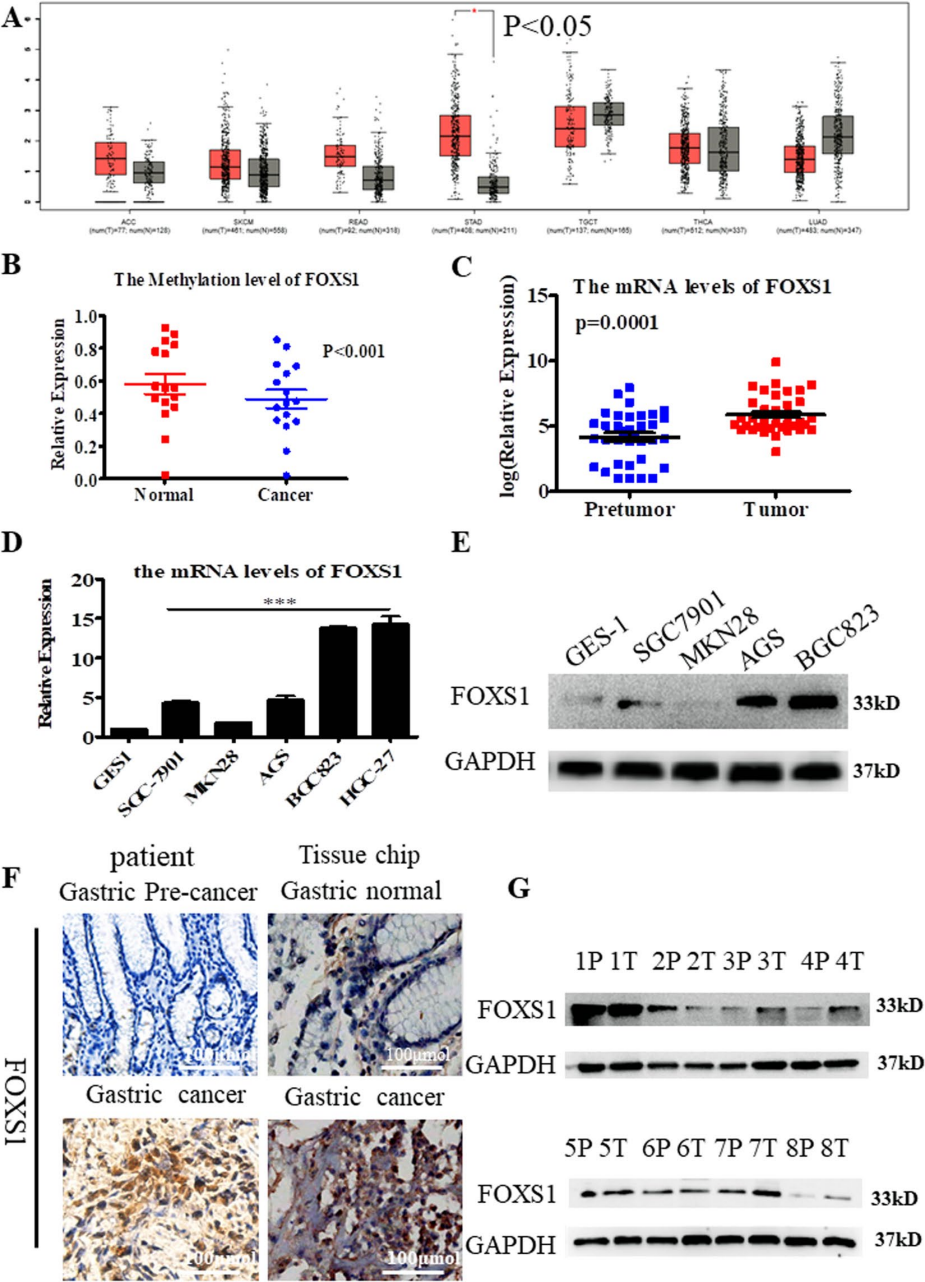
FOXS1 is high expression in gastric cancer. (**A**) FOXS1 expression levels in Adrenocortical carcinoma (ACC), skin cutaneous melanoma (SKCM), Stomach adenocarcinoma (STAD), Rectum adenocarcinoma (READ), Thyroid carcinoma (THCA), Testicular Germ Cell Tumors (TGCT), Thyroid carcinoma (THCA) and Lung adenocarcinoma (LUAD) using GEPIA online software. (**B**) The methylation levels of FOXS1 gene analyzing by TCGA sequencing data. (**C**) The mRNA levels of FOXS1 were analyzed by RT-PCR in 35 pairs of gastric cancer and adjacent nontumor tissues (n = 35). (**D**) The mRNA levels of FOXS1 were analyzed by RT-PCR in six kinds of gastric cancer cells (SGC7901, MKN28, AGS, BGC823 and HGC-27) and one gastric normal epithelial cell GES-1. (**E**) The protein levels of FOXS1 were analyzed by WB in four kinds of gastric cancer cells (SGC7901, MKN28, AGS and BGC823) and one gastric normal epithelial cell GES-1. (**F**) Left, immunohistochemical staining of FOXS1 in one paired samples of gastric cancer versus adjacent normal tissues; Right, immunohistochemical staining of FOXS1 in one sample of gastric cancer and non-adjacent gastric cancer normal tissues. (**G**) The protein levels of FOXS1 in 8 paired samples of gastric cancer versus adjacent normal tissues.

To examine the expression of FOXS1 in gastric cancer, we found that FOXS1 was significantly highly expressed in gastric cancer, but not in six other kinds of cancers by using GEPIA online software (Fig. 1A). By analyzing the cancer genome atlas (TCGA) data, we found that FOXS1 expression was significantly higher in gastric cancer samples than in noncancerous samples (normal = 38, cancer = 371, *P* < 0.001), consistent with the finding that FOXS1 expression was higher in in gastric cancer tissue than in normal gastric tissue in GSE19826 (N = 15, C = 12, *P* < 0.001), GSE13911 (N = 31, C = 38, *P* < 0.001) and GSE51575 (N = 26, C = 26, *P* < 0.001) datasets (Table 1). Using genome-wide methylation studies we identified lower FOXS1 methylation in gastric cancer, additional evidence indicating that FOXS1 is more highly expressed in gastric cancer tissues than in normal gastric tissues (*P* < 0.001, Fig. 1B). To validate the TCGA analysis results, we measured the expression of FOXS1 in 35 paired gastric cancer samples and paired precancer samples. The RT-PCR results showed that FOXS1 expression is significantly higher in gastric cancer tissues than in precancer tissues (*P* = 0.001, Fig. 1C). In addition, FOXS1 expression was significantly higher in gastric cancer cells than in GES-1 cells (normal gastric epithelial cell) at both the transcriptional (*P* < 0.001, Fig. 1D) and translational (Fig. 1E) levels. The immunohistochemistry (IHC) results also showed that FOXS1 expression was significantly higher in gastric cancer tissues than in the corresponding precancer tissues (Fig. 1F), consistent with the western blot (WB) analysis results shown in Fig. 1G. To further verify this result, IHC was performed on the tissue microarray chips containing 15 samples of early-stage, 55 samples of advanced-stage GC and 10 normal gastric tissue samples. The results showed that the average FOXS1 expression level was higher in 7 of the 15 samples of early-stage GC, and 45 of the 55 samples of advanced-stage GC than in the 10 normal tissues relative to the average expression of 10 normal tissues (Supplementary Figs S1–S3). These results suggest that FOXS1 expression can be a unique indicator of advanced gastric cancer.

**Table 1:** The FOXS1 gene expression in GEO data and TCGA data.

FOXS1	GSE19826		GSE13911		GSE51575		TCGA	
	N15	C12	N31	C38	N26	C26	N38	C371
logFC	1.74		0.645252		1.85		1.341	
P value	3.64E-04^***^		3.77E-02^***^		8.52e-08^***^		<0.001^***^	

^***^P < 0.001 were considered statistically significant.

### High FOXS1 expression indicates poor prognosis in gastric cancer patients

**Figure 2:**
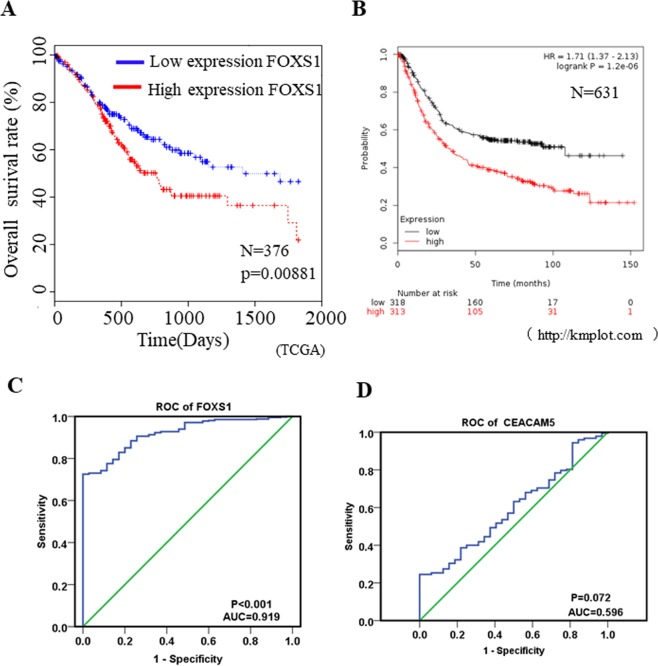
High expression of FOXS1 indicates poor prognosis for gastric cancer patients (**A**) Kaplan-Meier curves of patients with gastric cancer with low versus high expression of FOXS1 using TCGA sequencing data (N = 371; *P* = 0.00881). (**B**) Kaplan-Meier curves of patients with gastric cancer with low versus high expression of FOXS1 using online software Kaplan-Meier Plotter (http://kmplot.com) (N = 631; *P* < 0.001). (**C**) ROC curve of FOXS1was determined by SPSS and the AUC was 0.919, *P* < 0.001. (**D**) ROC curve of CEACAM5 was determined by SPSS and the AUC was 0.596, *P* = 0.072.

To explore the clinicpathological role of FOXS1 in gastric cancer, we classified 376 patients into two groups on the basis of the mean FOXS1 expression level by analyzing TCGA data. Patients with higher FOXS1 expression had significantly poorer prognoses than those with lower FOXS1 expression (P = 0.0081, Fig. 2A). The prognostic significance of FOXS1 mRNA assessed by the KM plotter application (http://kmplot.com/analysis/)^19^ also showed that upregulated FOXS1 expression was significantly correlated with poor survival and exhibited clear prognostic trend in 631 gastric cancer patients (*P* < 0.001, HR = 1.9; Fig. 2B). In addition, chi-square test showed that upregulated FOXS1 expression was correlated with patient age (P = 0.016), and T stage (P = 0.001). However, no significant differences were observed regarding patient sex, N stage, M stage, FIGO stage and the presence of residual tumor (Table 2). To assess whether FOXS1 could be used to predict gastric cancer development, we applied univariate and multivariate analyses. The univariate analysis results indicated that T stage (*P* = 0.023), N stage (*P* = 0.038), M stage (*P* = 0.003), age (*P* = 0.017), FIGO stage(I + II/III + IV) (*P* = 0.002) and FOXS1 expression (*P* = 0.009) influenced the survival rate of gastric cancer patients (Table 3), while the results of multivariate analysis applying a Cox proportional hazards model indicated that only age (*P* = 0.001), M stage (*P* = 0.022) and FOXS1 expression (*P* = 0.009) were independent impact factors for gastric cancer patients (Table 3). Furthermore, to evaluate the usefulness of FOXS1 in discriminating gastric cancer patients from healthy persons, we performed receiver-operating characteristic (ROC) analysis. The ROC analysis results revealed that FOXS1 could be a valuable biomarker for distinguishing gastric cancer patients from healthy persons, with an area under the curve (AUC) of 0.919 (Fig. 2C). Notably, the diagnostic value. of CEACAM5, a diagnostic marker for gastric cancer at the protein level that has frequently been used in the clinic^20^, for distinguishing gastric cancer patients from healthy persons was much lower (AUC = 0.596, *P* = 0.072) than that of FOXS1 (Fig. 2D). These results imply that FOXS1 exhibits powerful prognostic and diagnostic value for gastric cancer patients and can be a potential therapeutic target in gastric cancer.

**Table 2:** The correlation between FOXS1 gene expression level and patients’ clinical parameter.

	Number		FOXS1 expression Low / High		*P-*value
gender	female	148	78	70	0.392
	male	267	129	138	
age	< = 65	182	78	104	0.016^**^
	> = 65	244	123	101	
Stage(T)	1/2	110	69	41	<0.001^***^
	3/4	296	131	165	
Stage(N)	0	124	66	58	0.270
	1/2/3	273	129	144	
Stage(M)	0	367	183	184	0.842
	1	27	14	13	
Ajcc	1/2	181	92	89	0.559
	3/4	211	101	110	
residual tumor	Free	330	161	169	0.077
	With	34	22	12	

Low/high by the sample mean. Pearson’s Chi-square tests.

^**^P < 0.01 and ^***^P < 0.001 were considered statistically significant.

**Table 3:** Univariate and multivariate analyses of the contribution of FOXS1 on the survival of GC patients.

Clinical parameters	Univariate analysis		Multivariate analysis	
	HR^a^ (95%CI)	*P* value	HR^a^ (95%CI)	*P* value
FOXS1	1.001 (1.000–1.003)	0.009^**^	1.002 (1.000–1.003)	0.009^**^
Age (years)	1.022 (1.004–1.041)	0.017^**^	1.033 (1.014–1.052)	0.001^**^
T stage (T1 + T2/T3 + T4)	1.306 (1.038–1.643)	0.023^*^	1.203 (0.931–1.555)	0.158
N stage (No/Yes)	1.259 (1.013–1.565)	0.038^*^	1.014 (0.732–1.404)	0.934
M stage (No/Yes)	1.501 (1.153–1.955)	0.003^**^	1.433 (1.095–1.876)	0.009^**^
Stage(I + II/III + IV)	1.356 (1.119–1.642)	0.002^**^	1.254 (0.927–1.696)	0.143
Grade(I + II/III + IV)	1.194 (0.986–1.446)	0.069	—	—
Tumor size(< = 10 mm/>10 mm)	3.182 (1.614–2.671)	<0.001^***^	—	—
Gender	1.467 (0.978–2.199)	0.064	—	—

^a^HR, hazad ratio. ^*^P < 0.05, ^**^P < 0.01, ^***^P < 0.001 were considered statistically significant.

### FOXS1 regulates gastric cancer cell proliferation and colony formation

**Figure 3:**
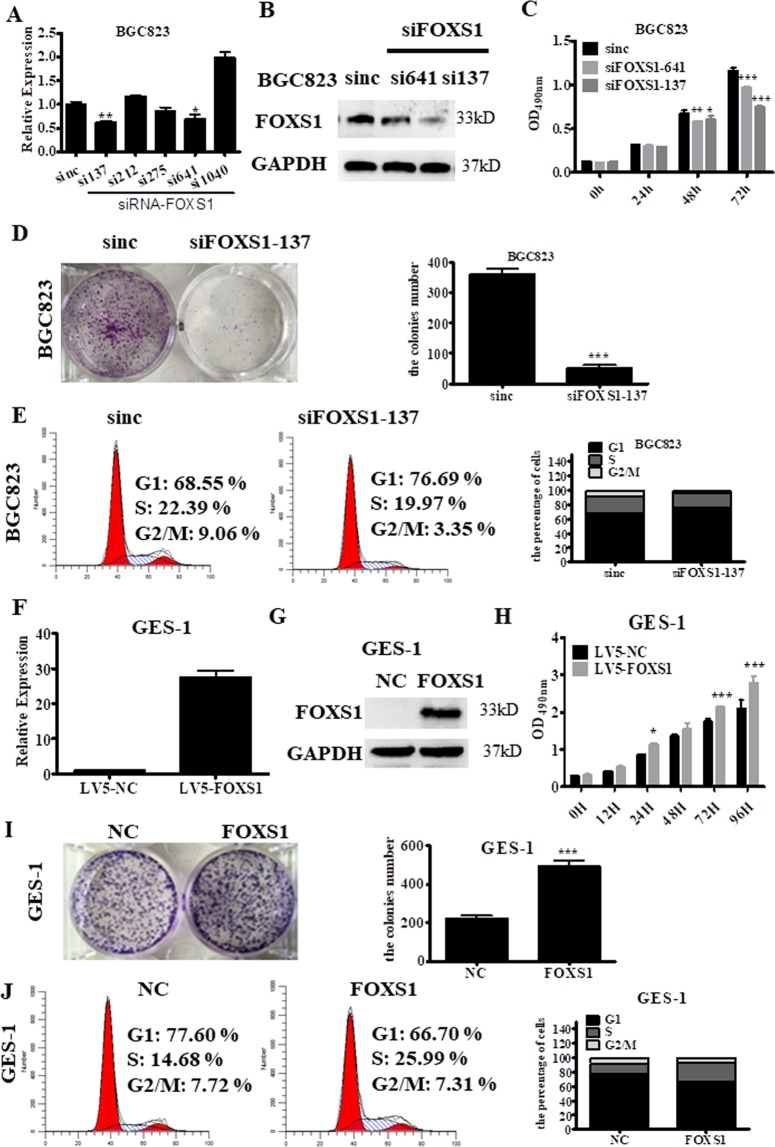
FOXS1 regulates gastric cancer cell proliferation and the ability of colony formation. (**A**) The knockdown effect of siRNA targeting at five sites (137, 212, 275, 641 and 1040) of FOXS1 mRNA at mRNA levels were detected by RT-PCR. (**B**) The knockdown effect of siRNA targeting at two sites (137 and 641) of FOXS1 mRNA at protein levels were detected by WB. (**C**) The effect of FOXS1 knockdown by transfection with siRNA-137 or siRNA-641 targeting FOXS1 on cell viability was determined by Cell Proliferation Assay kits. (**D**) The effect of FOXS1 knockdown by transfection with siRNA-137 targeting FOXS1 on cell colony formation was analyzed by Crystal violet staining, and quantitative analysis of cell colony numbers was showed in the right panel. (**E**) The effect of FOXS1 knockdown on cell cycle distribution. (**F**,**G**) Overexpression effect of FOXS1 at mRNA levels was confirmed by RT-PCR and at protein levels was confirmed by WB. (**H**) Effect of FOXS1 overexpression on cell viability were detected by Cell Proliferation Assay kits. (**I**) The effect of FOXS1 overexpression on cell colony formation was analyzed by Crystal violet staining, and quantitative analysis of cell colony numbers was showed in the right panel. (**J**) The effect of FOXS1 overexpression on cell cycle distribution.

To clarify the functions of FOXS1, we designed five independent short interfering RNAs (siRNAs) targeting FOXS1 and transfected these siRNAs into BGC823 cells, which exhibit a relatively high level of FOXS1expression. The results showed that targeting FOXS1 with siRNA-137 or siRNA-641 effectively knocked down FOXS1 expression in BGC823 cells at both mRNA level (P < 0.01, P < 0.05, respectively; Fig. 3A) and the protein level (Fig. 3B). Cell proliferation assays revealed that silencing FOXS1 by transfection with siRNA-137 or siRNA-641 targeting FOXS1 significantly inhibited cell proliferation relative to that of the control cells (Fig. 3C). In addition, FOXS1 silencing significantly reduced the colony numbers in BGC823 cells (P < 0.001, Fig. 3D). Moreover, the flow cytometry (FCM) results showed that blocking FOXS1 expression in BGC823 cells led to an accumulation of cells in G1 phase and a decrease in the number of cells in the S and G2/M phases (Fig. 3E). In contrast, stable overexpression of FOXS1 in GES-1 cells and SGC7901 cells, which exhibit a relatively low level of FOXS1expression, significantly promoted cell proliferation (Fig. 3F–H and Supplementary Fig. S4A–C) and increased the colony numbers (Fig. 3I and Supplementary Fig. S4D). The flow cytometry (FCM) results further revealed a reduction in the G1-phase population and an increase in the S-phase population of GES-1 cells overexpressing FOXS1 (Fig. 3J). These data demonstrate that FOXS1 affects the G1-S transition in cell-cycle progression and  increases the proliferative and colony-forming abilities of GC cells.

### FOXS1 promotes gastric cancer cell migration, invasion and EMT

**Figure 4:**
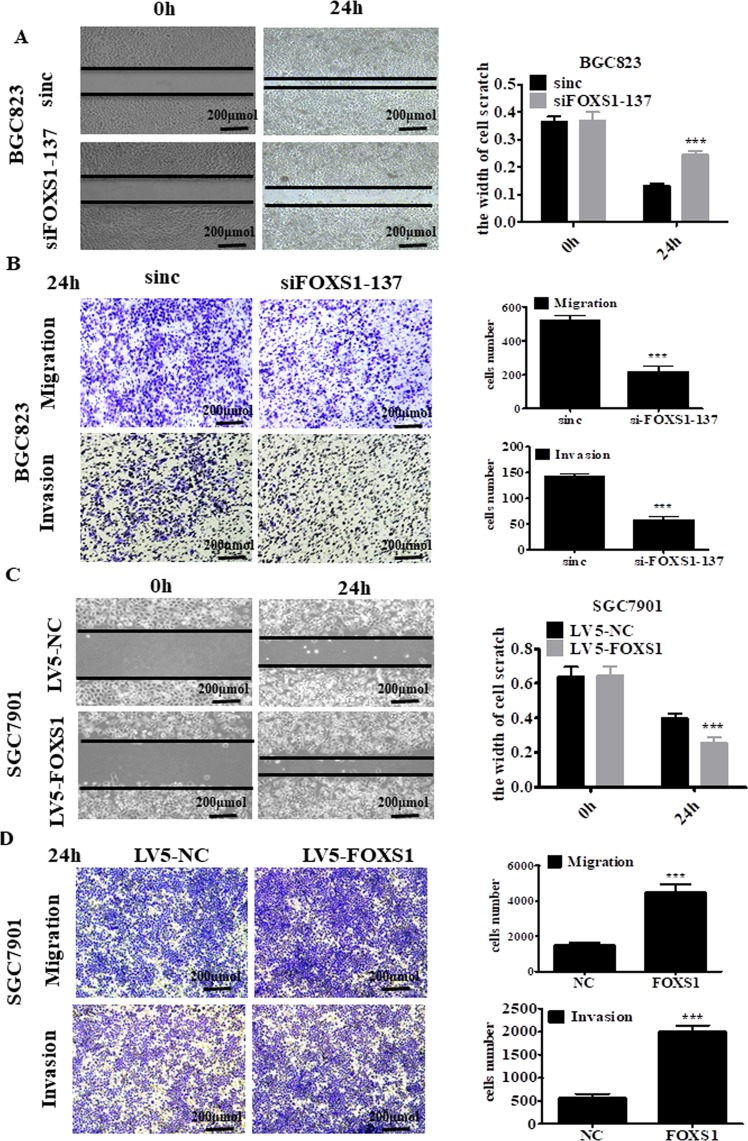
FOXS1 promotes gastric cancer cell migration and invasion *in vitro*. (**A**,**C**) Effect of FOXS1 knockdown or FOXS1 overexpression on the wound-healing process in indicated cells and the scratch width were quantitative analyzed in the right panel. (**B**,**D**) Effect of FOXS1 knockdown or FOXS1 overexpression on metastatic ability of gastric cancer cells using *in vitro* migration and invasion transwell assays. Statistical analysis has shown in the right panel.

**Figure 5:**
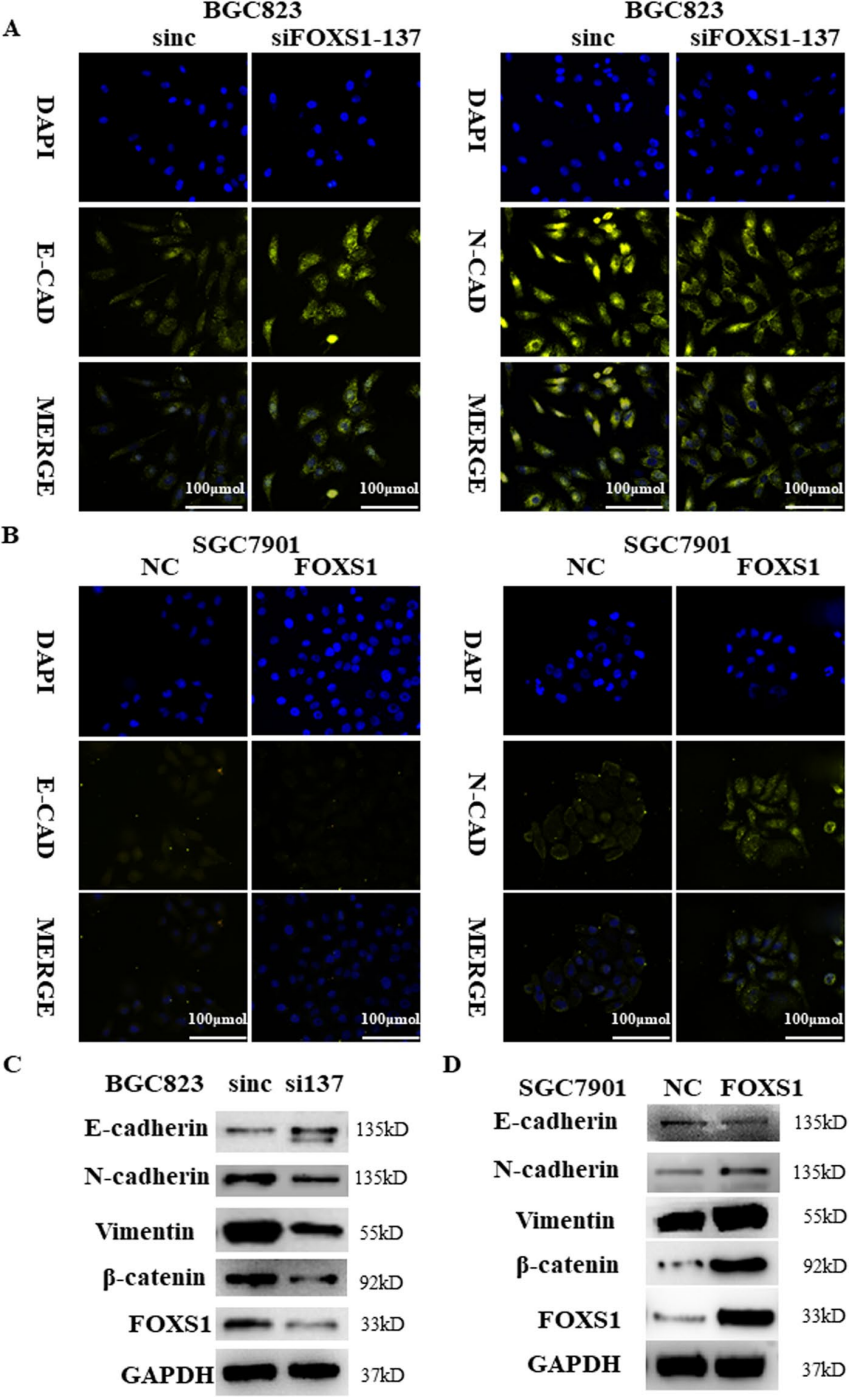
FOXS1 promotes gastric cancer cell EMT *in vitro*. (**A**,**B**) The influence of FOXS1 knockdown or overexpression on EMT markers (E-cadherin and N-cadherin) were detected by immunofluorescence. (**C**,**D**) The influence of FOXS1 knockdown or overexpression on the proteins (E-cadherin, N-cadherin, Vimentin, β-catenin and GAPDH) were detected by WB.

We further investigated the effect of FOXS1 on the migration and invasive abilities of gastric cancer cells using *in vitro* Transwell assays with or without a Matrigel matrix layer on the inserts. Knockdown of FOXS1 in BGC823 cells significantly suppressed the cell wound healing, migration and invasive abilities (Fig. 4A,B), while FOXS1 overexpression in SGC7901 cells significantly increased cell wound healing, migration and invasive abilities (Fig. 4C,D). To further prove the effect of FOXS1 on invasion and migration in gastric cancer, we performed WB analysis and immunofluorescence to measure the expression levels of EMT markers. The immunofluorescence results showed that FOXS1 knockdown increased the level of the epithelial marker E-cadherin (Fig. 5A left), but decreased the levels of the mesenchymal marker N-cadherin (Fig. 5A right). As predicted, FOXS1 overexpression produced the opposite results (Fig. 5B). Consistent with the above results, the WB analysis results showed that FOXS1 knockdown significantly inhibited the expression of N-cadherin, Vimentin and β-catenin, but increased E-cadherin expression. However, FOXS1 overexpression produced the inverse results (Fig. 5C,D). To further determine whether FOXS1 promotes EMT via the Wnt/β-catenin pathway, we next detected the expression of Wnt/β-catenin pathway related proteins (such as Cyclin-D1, and c-Myc)^21^. The RT-PCR results showed that FOXS1 overexpression significantly enhanced the gene expression of Cyclin-D1and c-Myc (Supplementary Fig. S5).

### FOXS1 promotes tumor growth and EMT *in vivo*

**Figure 6:**
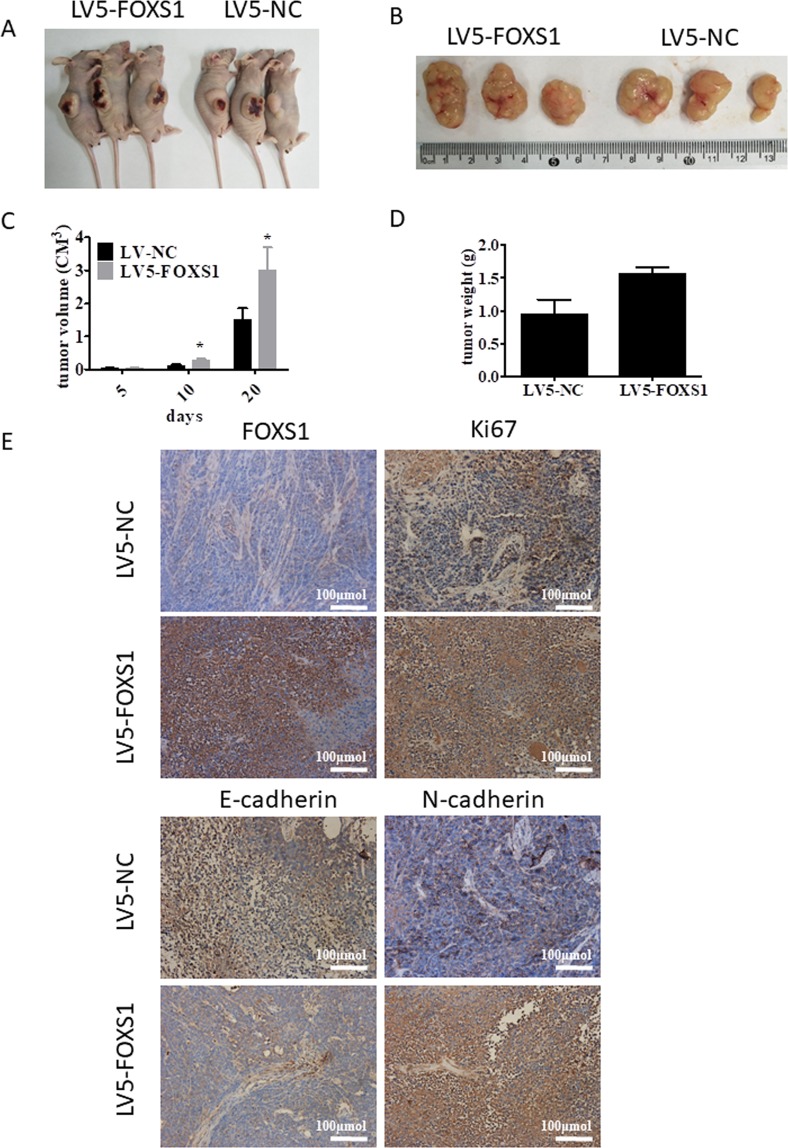
FOXS1 promotes gastric cancer cell growth and altered the expression of EMT markers *in vivo*. (**A**) The nude mouse models with FOXS1 xenografts was successful developed by respectively injected SGC7901 cells infected with LV5-FOXS1 or LV-NC into each flank of nude mice. (**B**) A representative picture of the morphology of tumor xenografts after excision at 20 days of treatment. (**C**) FOXS1 overexpression accelerated tumor growth in nude mice. (**D**) The tumor weight in LV-FOXS1 group and LV-NC control group were quantitatively analyzed. (**E**) The FOXS1 expression in the FOXS1-infected tumors was confirmed by IHC staining. Cell proliferation in tumors isolated from FOXS1-overexpressing or control nude mice xenografts were determined by Ki-67 staining. EMT markers E-cadherin was decreased in FOXS1-infected tumors while N-cadherin expression was enhanced in FOXS1-infected tumors.

To further confirm whether FOXS1 could affect tumorigenesis and EMT in gastric cancer *in vivo*, SGC7901 cells infected with LV5-FOXS1 or control lentivirus were injected into the flank of nude mice. As shown in Fig. 6A, the indicated gastric cancer cells successfully formed gastric cancer tumors and the tumors shown in Fig. 6B were collected and measured. The results showed that FOXS1 overexpression increased tumor growth in terms of both size and weight in nude mice (Fig. 6C,D). The IHC results showed that FOXS1 was highly expressed in the LV5-FOXS1-infected SGC7901 cells, indicating that the LV5-FOXS1 lentivirus was successfully transduced into SGC7901 cells (Fig. 6E). In addition, positive Ki67 and N-cadherin staining was much stronger in FOXS1 overexpressing SGC7901 cells than in control cells, while E-cadherin expression was lower in FOXS1 overexpressing SGC7901 cells than in control cells (Fig. 6E). These *in vivo* data further demonstrated that FOXS1 can promote gastric cancer tumorigenesis and EMT events.

### Analysis and identification of the FOXS1 promoter region and transcription factors

**Figure 7:**
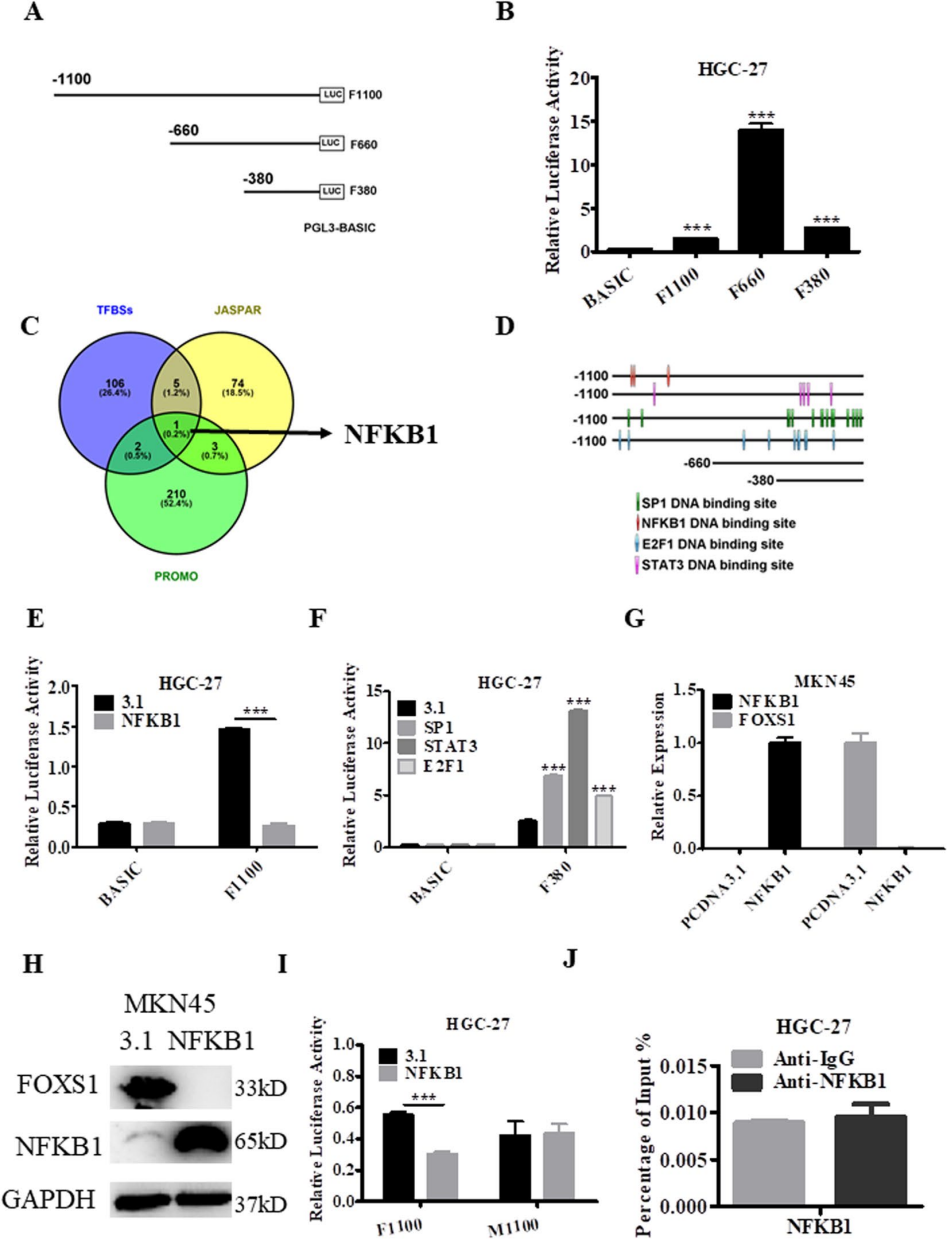
The analysis and identification of FOXS1 core promoter. (**A**) Schematic diagram of FOXS1 upstream 1100 bp promoter reporter constructs. (**B**) Dual luciferase reporter assays were used to determine the core promoter activity region of FOXS1. (**C**,**D**) Prediction of transcription factors binding sites in the FOXS1promoter region using TFBSs, JASPAR and PROMO. (**E**,**F**) The influence of transcription factors on FOXS1 promoters were determined by dual luciferase reporter assays. (**G**) The expression of NFKB1 and FOXS1 after NFKB1 overexpression was determined by RT-PCR in MKN45 cells. (**H**) The expression of NFKB1 and FOXS1 after NFKB1 overexpression was determined by western blot in MKN45 cells. GAPDH was internal control. (**I**) The effect of transcription factors NFKB1 on FOXS1 promoters with NFKB-binding mutated sites were determined by dual luciferase reporter assays. (**I**) ChIP assays were performed to detect NFKB1 directly bound in the FOXS1 promoter region in HGC-27 cells.

In the UCSC database, the promoter region of FOXS1was predicted to be located at chr20: 31,845,488-31,846,588, and three luciferase reporter vectors containing the indicated genomic fragments of the FOXS1gene were constructed (Fig. 7A). The results of the dual-luciferase assay showed that the luciferase activity was significantly increased in HGC27 cells transfected with F1100, F660 and F380 compared with that in HGC-27 cells transfected with the pGL3-Basic vector. In addition, the genomic region from nucleotides −660 to +1 in the FOXS1 gene had stronger promoter activity than other regions in this gene (Fig. 7B). To further investigate the potential regulators involved in FOXS1 expression, potential transcription factor binding sites in the FOXS1 promoter were identified using three online software packages: JASPAR (http://jaspar.genereg.net/), TFBSs (http://biogridlasagna.engr.uconn.edu/lasagna_search/) and PROMO (https://wwwbimas.cit.nih.gov/molbio/proscan/). As shown in Fig. 7C, NFKB1 binding sites were found in the promoter region of FOXS1 in all three independent databases. In addition, binding sites for the other three transcription factors SP1, STAT3 and E2F1 were analyzed by JASPAR along with the NFKB1 transcription factor binding sites. The results showed that the 3 potential NFKB1-binding sites were mostly located in the region between nucleotides −1100 ~ −660 in FOXS1 genes, while the 11 potential SP1-binding sites, the 4 potential STAT3-binding sites and the 7 potential E2F1-binding sites were mostly located in the region between nucleotides −380 ~ +1 in the FOXS1 genes (Fig. 7D). NFKB1 binding significantly inhibited luciferase activity (Fig. 7E), while SP1, STAT3 and E2F1 binding markedly enhanced luciferase activity driven by the promoter regions of the FOXS1 gene (Fig. 7F). These results led us to hypothesize that NFKB1 may function as a transcriptional repressor but SP1, STAT3 and E2F1 act as transcriptional activators coregulating FOXS1 expression. To further investigate whether exogenous NFKB1 negatively regulates FOXS1, an NFKB1 overexpression plasmid was transfected into MKN45 cells. The results showed that exogenous NFKB1 overexpression significantly inhibited FOXS1 expression at both the mRNA (Fig. 7G) and protein (Fig. 7H) levels, suggesting that exogenous NFKB1 may be an effective treatment strategy targeting FOXS1 in GC patients. Moreover, the NFKB1 binding motifs “GTGGAT**T**TCC” in the promoter-reporter constructs were mutated to “GTGGAT**G**TCC” in the promoter-reporter constructs of FOXS1 and the results showed that the effect of NFKB1 overexpression on luciferase activity driven by the F1100 region after mutation of NFKB1 binding motif sequence was abrogated (Fig. 7I). A further ChIP assay was performed and the results showed that NFKB1 can’t directly bound to the FOXS1 promoter region (Fig. 7J), indicating that NFKB1 deregulate the expression of FOXS1 in other special ways.

### The FOXS1 gene was most abundantly enriched in the Hh pathway and was identified as a GLI1 target gene in GC

**Figure 8:**
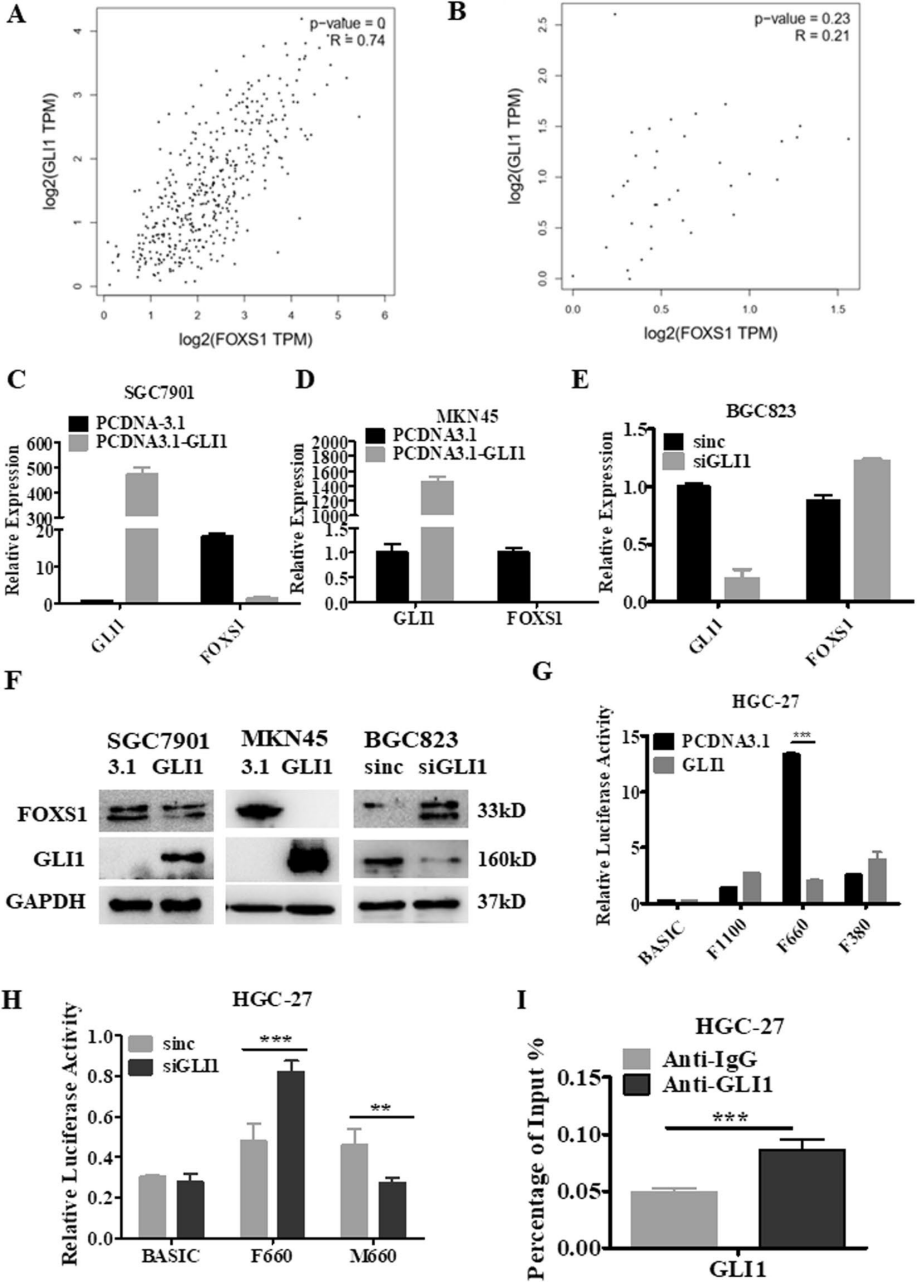
FOXS1 expression positively correlates with GLI1 expression and FOXS1 is a target gene of GLI1. The pearson correlation between FOXS1 and GLI1 expression was detected by analyzing TCGA data in STAD tumor samples (**A**) and in STAD normal samples (**B**). FOXS1 and GLI1 expression was detected by RT-PCR in SGC7901 (**C**) and MKN45 cells (**D**) transfected with pCDNA3-vector or pCDNA3-GLI1. (**E**) FOXS1 and GLI1 expression was detected by RT-PCR in BGC823 cells transfected with siRNA control or siRNA targeting GLI1. (**F**) The protein levels of FOXS1 and GLI1 was detected by western blot in SGC7901 and MKN45 cells transfected with pCDNA3-vector or pCDNA3-GLI1 or in BGC823 cells transfected with siRNA control or siRNA targeting GLI1. (**G**) Dual luciferase activity analysis of the four promoter activity regions of FOXS1 after transfection with pCDNA3-vector or pCDNA3-GLI1. (**H**) Dual luciferase activity analysis of the F660 and M660 promoter of FOXS1 after GLI1 knockdown. M660 refers to core promoter reporting vectors with GLI1 binding motif mutation sites. (**I**) ChIP assays were performed to detect GLI1 directly bound in the FOXS1 promoter region in HGC-27 cells.

The RNA-seq data for patients in the TCGA database were used to divide patients into the FOXS1 high expression group and the FOXS1 low expression group according to the mean level of FOXS1 gene expression. Enrichment analysis was performed on the two groups of genes to screen statistically significant signaling pathways by GSEA (software http://software.broadinstitute.org/gsea/index.jsp). We found that the FOXS1 gene was significantly enriched in the KEGG_HEDGEHOG_SIGNALING_PATHWAY, KEGG_ECM_RECEPTOR_INTERACTION, KEGG_FOCAL_ADHESION, KEGG_VASCULAR_ SMOOTH MUSCLE_CONTRACTION and 11 other important signaling pathways (Table 4). Notably, FOXS1 was most abundantly enriched in the Hh signaling pathway (NES, 0.61; FDR, q = 0; FWER, p = 0.003). In GC, tumorigenicity and EMT are regulated through the activation of GLI1, a key member of the Hh signaling pathway^13^. Importantly, correlation analysis revealed that the expression of FOXS1 was strongly correlated (R = 0.74, *P* < 0.001) with the expression of GLI1 in the STAD tumor dataset (Fig. 8A), whereas FOXS1 expression was not correlated with GLI1 expression in the TCGA STAD normal dataset (Fig. 8B), indicating an interplay between GLI1 and FOXS1 expression in STAD tumors. Next, we determined the expression of GLI1 in three kinds of gastric cancer cells and GES-1 cells. The results showed that GLI1 had a high expression level in BGC823 cells, a moderate expression level in MKN45 and SGC7901 cells and a low expression level in GES-1 cells (Supplementary Fig. S6), an expression trend consistent with that of FOXS1. To further investigate whether FOXS1 is a target gene of GLI1 in STAD tumors, GLI1 exogenous overexpression and gene silencing experiments were performed. GLI1 overexpression significantly inhibited FOXS1 expression in both SGC7901 cells and MKN45 cells (Fig. 8C,8D and 8F left and middle), while GLI1 gene silencing upregulated FOXS1 expression in BGC823 cells (Fig. 8E and 8F right). Therefore, we hypothesized that the transcription factor GLI1 may be a transcriptional repressor of FOXS1. To investigate the potential GLI1/FOXS1 regulatory mechanism, dual luciferase assays were performed. Although GLI1 exerted a differential effect on luciferase activity driven by the FOXS1 promoter regions in HGC-27 and SGC7901 cells, GLI1 overexpression significantly decreased luciferase activity driven by the FOXS1 promoter at the core promoter region of F660 in both HGC-27 cells (Fig. 8G) and SGC7901 cells (Supplementary Fig. S7), while GLI1 silencing notably increased luciferase activity driven by the F660 region in HGC-27 cells (Fig. 8H). Moreover, we mutated the GLI1 binding motif “CAC**C**ACCCAG” to “CAC**A**ACCCAG” and found that the effect of GLI1 silencing on luciferase activity driven by the F660 region was reversed (Fig. 8H). A further ChIP experiment demonstrated that GLI1 directly bound to the FOXS1 promoter region (Fig. 8I). These findings indicate that GLI1 acts as a unique transcriptional repressor of FOXS1 by binding FOXS1 promoter regions in GC cells.

**Table 4:** Statistically significant signaling pathway for FOXS1 gene enrichment.

GS Details	NES	FDR q-val	FWER p-val
KEGG_HEDGEHOG_SIGNALING_PATHWAY	0.61	0	0.003**
KEGG_ECM_RECEPTOR_INTERACTION	0.67	0	0.005**
KEGG_FOCAL_ADHESION	0.56	0	0.006**
KEGG_VASCULAR_SMOOTH_MUSCLE_CONTRACTION	0.55	0	0.008**
KEGG_BASAL_CELL_CARCINOMA	0.58	0	0.007**
KEGG_NEUROACTIVE_LIGAND_RECEPTOR_INTERACTION	0.52	0	0.006**
KEGG_GAP_JUNCTION	0.5	0	0.009**
KEGG_CELL_ADHESION_MOLECULES_CAMS	0.56	0.004	0.015*
KEGG_DILATED_CARDIOMYOPATHY	0.55	0.004	0.021*
KEGG_CYTOKINE_CYTOKINE_RECEPTOR_INTERACTION	0.5	0.004	0.035*
KEGG_MELANOGENESIS	0.46	0	0.034*
KEGG_CALCIUM_SIGNALING_PATHWAY	0.47	0.004	0.042*
KEGG_HYPERTROPHIC_CARDIOMYOPATHY_HCM	0.52	0.006	0.044*
KEGG_REGULATION_OF_ACTIN_CYTOSKELETON	0.42	0.002	0.047*

^*^P < 0.05, ^**^P < 0.01, ^***^P < 0.001 were considered statistically significant.

### miR-125a-5p downregulated FOXS1 expression through translational repression

**Figure 9:**
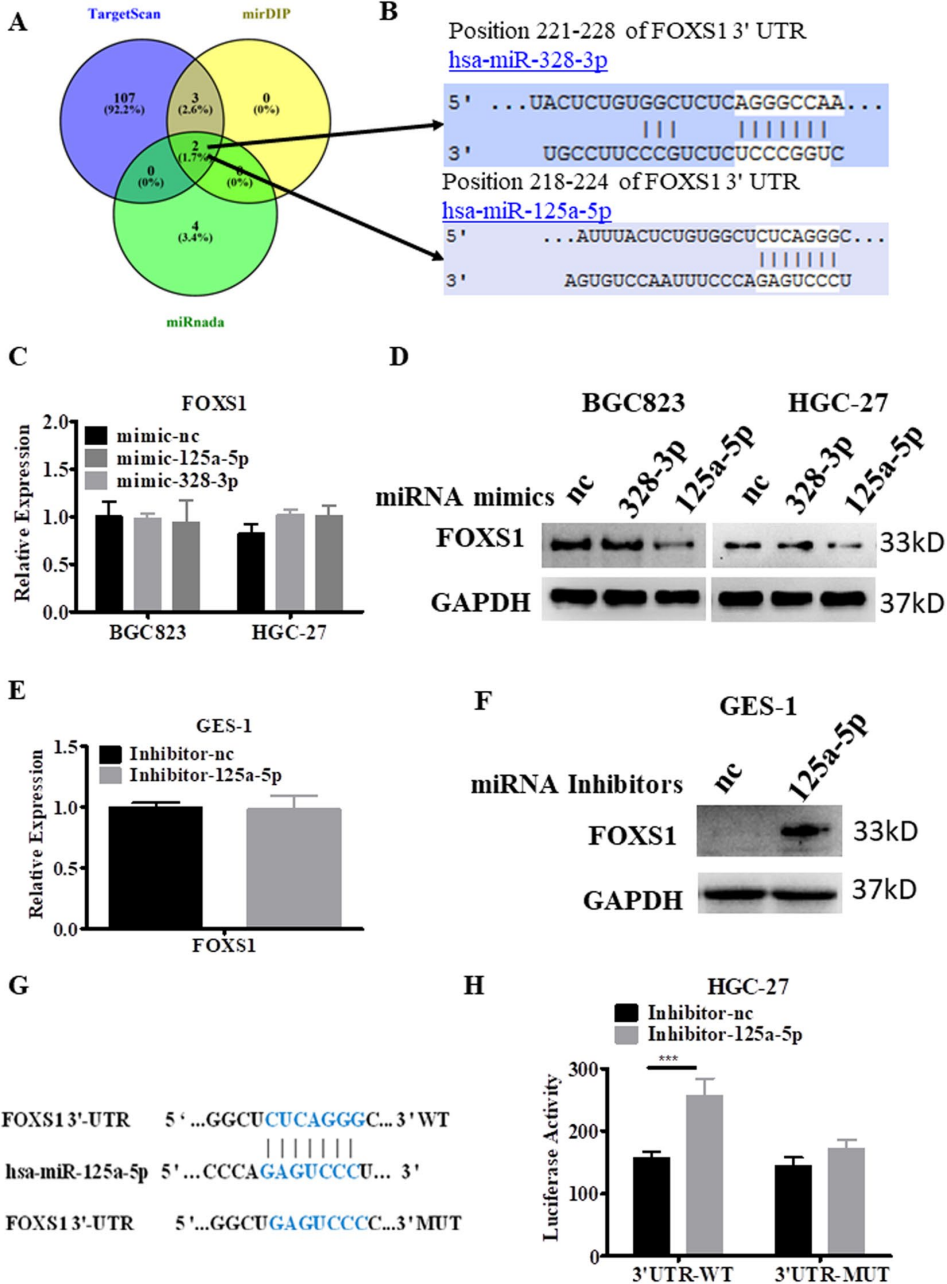
MiR-125a-5p inhibits FOXS1 expression by translational repression. (**A**) Three independent miRNA target databases were used to predict the potential miRNAs. (**B**) Schematic representation of the potential miRNAs binding sites in 3′UTR of FOXS1. (**C**) RT-PCR results of the expression of FOXS1 in the miRNA mimics of miR-328-3p or miR-125a-5p transfection group in BGC823 and HGC-27cells. (**D**) Levels of FOXS1 protein were analyzed by WB after transfection with mimics of miR-328-3p or miR-125a-5pfor 72 hours. (**E**) RT-PCR results of the expression of FOXS1 in GES-1 cells transfected with miRNA inhibitors of miR-125a-5p or nc. (**F**) Levels of FOXS1 protein were analyzed by WB after transfection with inhibitors of miR-125a-5p for 72 hours. (**G**) Predicted binding sites and mutant binding site between miR-125a-5p and 3′UTR OF FOXS1. (**H**) HGC-27 cells were firstly transfected with miR-125a-5p inhibitors or inhibitors nc and next day transfected with a luciferase reporter-containing FOXS1 wild-type 3′UTR (3′UTR-WT) or FOXS1 mutant 3′UTR (3′UTR-MUT). Luciferase activity of the indicated cells were determined by Luciferase assay kits.

To investigate the miRNA-related mechanism of FOXS1 upregulation in gastric cancer, we used 3 independent databases miRanda, mirDIP, and TargetScan to computationally predict miRNAs that may be involved (Fig. 9A). Two miRNAs (miR-328-3p and miR-125a-5p) were identified in all three independent datasets (Fig. 9B). Then, two miRNA mimics of these miRNAs were transfected into BGC823 and HGC27 cells, which exhibit high expression of FOXS1. The RT-PCR results showed that although miR-328-3p and miR-125a-5p were successfully overexpressed (Supplementary Fig. S8A,B), no difference in the mRNA levels of FOXS1 were detected after transfection of the miR-328-3p or miR-125a-5p mimics (Fig. 9C). However, the protein level of FOXS1 was appreciably decreased in miR-125a-5p-transfected cells compared with that in negative control-transfected cells, while no difference was found in miR-328-3p-transfected cells (Fig. 9D). To confirm the above conclusion, miR-125a-5p inhibitors were synthesized and transfected into GES-1 cells, which exhibit high levels of miR-125a-5p expression^22^ and low levels of FOXS1 expression. The RT-PCR results showed that the expression of miR-125a-5p was inhibited after transfection with miR-125a-5p inhibitors, with no change in the mRNA levels of FOXS1 (Fig. 9E and Supplementary Fig. S8C). As shown in Fig. 9F, the FOXS1 protein levels were markedly upregulated after transfection with miR-125a-5p inhibitors in the normal gastric cell line GES1. To investigate whether FOXS1 was a direct target of miR-125a-5p, wild-type or mutant binding site fragments in the FOXS1 3′ UTR (Fig. 9G) were directly fused downstream of the firefly luciferase gene in the GP-miRGLO vector. Cotransfection of miR-125a-5p inhibitors with the GP-miRGLO-FOXS1-3′UTR wild-type luciferase reporter plasmid caused a marked increase in luciferase activity, whereas luciferase activity was not significantly affected by cotransfection of miR-125a-5p inhibitors with the GP-miRGLO-FOXS1-3′UTR mutant luciferase reporter plasmid (Fig. 9H). Collectively, these results suggest that miR-125a-5p directly targets FOXS1 in gastric cancer cells, deregulating FOXS1 expression through translational repression.

## Discussion

Recent evidence suggests that FOX gene family members are important in a wide spectrum of biological processes^4,23,24^. However, the role of FOXS1, a new member of the FOX gene family^25^, in cancer has not been reported to date. In this study, we concluded that FOXS1 was highly expressed in gastric cancer. Tissue microarray revealed that FOXS1could be a unique indicator of advanced gastric cance. In addition, high FOXS1 expression indicated poor survival and differentiated gastric cancer from normal tissues with high sensitivity and specificity. FOXS1 expression was positively related to tumor size and advanced age and could be an independent prognostic factor. In addition, FOXS1 promoted cell proliferation, migration and invasion via the Wnt/β-catenin pathway, which may be coregulated by the transcriptional activators STAT3, SP1, and E2F1 and the transcriptional repressor NFKB1. Exogenous NFKB1 overexpression inhibited FOXS1 expression, suggesting a new treatment target for GC patients. In addition, FOXS1, identified as a target gene of GLI1, was most abundantly enriched in the Hh signal pathway. Finally, miR-125a-5p bound the 3′UTR of FOXS1 and regulated the expression of FOXS1 via translational repression in gastric cancer cells, which may lead to further advancements in the knowledge of gastric cancer tumorigenesis.

Given that Fox proteins control these essential developmental and homeostatic processes, a loss or gain of Fox function can alter cell fate and lead to tumorigenesis^4,26–28^. In our study, we found that FOXS1 was highly expressed in gastric cancer. In addition, FOXS1 overexpression in GES-1 and SGC7901 cells promoted cell proliferation and cell colony formation ability, while FOXS1 silencing in BGC823 produced the inverse results. These results are consistent with the report that FOXS1 and six other genes were positive impact factors serving as a prognostic signature to predict the survival of gastric cancer patients and to monitor the long-term treatment of gastric cancer^29^. Nevertheless, FOXS1 has the opposite characteristic in liver cancer; it is expressed at lower levels in most HCC tissues than in normal liver tissues. These decreased expression levels are correlated with tumor size, AJCC stage, and tumor differentiation, acting as an important factor for predicting the prognosis of HCC patients^30^. From the above results, we concluded that members of the FOX gene family, including FOXS1, can be oncogenes or tumor suppressor genes depending on the cancer type.

EMT also plays an important role in the invasion and metastasis of cancers^31^. As a result of EMT, tumor cells exhibit the characteristics of downregulated expression of epithelial-associated markers such as E-cadherin and upregulated expression of mesenchymal markers such as N-cadherin and Vimentin^13^. In our work, we found that FOXS1 expression was inversely correlated with the expression of epithelial markers (E-cadherin) and positively correlated with the expression of mesenchymal markers (vimentin and N-cadherin), indicating that FOXS1 could induce EMT in gastric cancer cells. A large body of evidence suggests that the canonical Wnt/β-catenin pathway plays an important role in inducing epithelial cancer cells to undergo EMT^32,33^. In our work, we found that FOXS1 silencing inhibited β-catenin expression while FOXS1 overexpression augmented β-catenin expression. In addition, wnt/β-catenin can regulate important tumor-related genes including c-Myc and Cyclin D1^21^. We investigated the effect of FOXS1 on the Wnt/β-catenin signaling pathway, and found that FOXS1 overexpression significantly enhanced the mRNA levels of c-Myc and Cyclin D1. These results preliminarily indicate that FOXS1 promotes EMT via the Wnt/β-catenin signaling pathway.

Despite the extensive efforts to understand the contribution of FOXS1 to gastric cancer, the mediators responsible for FOXS1 upregulation in gastric cancer remain unidentified. In our work, the results of luciferase reporter assays showed that the core promoter regions of FOXS1 are located at nucleotides −660~ +1 in gastric cancer HGC-27 cells, but at −380 ~ +1 in SGC7901 cells (Supplementary Fig. S9A). In addition, FOXS1 has 11 potential SP1-binding sites, 4 potential STAT3-binding sites, and 7 potential E2F1-binding sites, which are mostly located in the −380 ~ +1 region of the FOXS1 gene, and 3 NFKB1-binding sites in the −1100 ~ −660 region. As predicted, the luciferase reporter assay showed that STAT3, E2F1, and SP1 enhanced luciferase activity driven by F380 in both HGC-27 cells and SGC7901 cells. Unexpectedly, NFKB1 inhibited luciferase activity driven by F1100 in HGC-27 cells but slightly increased luciferase activity driven by F1100 in SGC7901 cells (Supplementary Fig. S9B), suggesting that NFKB1 regulates FOXS1 expression in a manner dependent on the type of gastric cancer cells.

Glioma-associated oncogene 1 (GLI1) is one of the three GLI transcription factors within the Hh pathway^34^. Yan and coworkers found that Gli1 overexpression is a frequent event in gastric cancer tissues^35,36^,and is correlated with characteristics of a more aggressive phenotype, including poorly differentiated histology, advanced TNM stage, serosal involvement and lymph node metastasis^35^. In this study, we found that FOXS1 expression was significantly correlated with GLI1 expression in the STAD tumor dataset but was not correlated with GLI1 expression in the STAD normal dataset, suggesting that GLI1 and FOXS1 may exhibit interplay in gastric cancer. Moreover, Diao *et al*. recently published that both GLI1 and FOXS1 are highly expressed and positively correlated with GLI1 in medulloblastoma samples^15^, further suggesting that the existence of FOXS1/GLI1 interplay in human cancers may not be dependent on the type of human tumor. Interestingly, FOXS1 expression can reduce GLI1 transcriptional activity, and GLI1/FOXS1 exhibit direct protein–protein interaction in HEK293A cells^15,37^. In our study, we found that GLI1 inhibited FOXS1 expression by binding the promoter regions of FOXS1, suggesting that GLI1 and FOXS1, two oncogenes, may combine and suppress each other’s expression. This phenomenon suggests that there may be negative feedback between FOXS1 and GLI1 in human cancers that needs to be further studied.

Mature miRNAs are a large group of conserved, single-stranded, noncoding RNAs of approximately 22 nucleotides (nt) in length that act as posttranscriptional regulators of gene expression by binding to their target coding mRNA through imperfect complementarity at multiple sites in the 3′-UTR of a gene, resulting in cleavage, translational repression, or chromatin modification of the coding mRNA and thus interfering with gene expression^38–44^. It is worth noting that low expression of miR-125a-5p was previously reported to be an independent prognostic factor in gastric cancer and inhibits the proliferation, invasion and metastasis of human gastric cancer cells^42^. In addition, Cao Y. *et al*. reported that miR-125a-5p expression was lower in human gastric cancer cell lines (SGC7901, HGC27 and BGC823) than in the normal gastric mucosa cell line GES1, with a similar pattern in 82 pairs of cancer tissues and matched normal tissues, as evidenced by RT-PCR analysis^22,42^, while FOXS1 was highly expressed in the above gastric cancer cell lines and gastric cancer tissues, revealing that the endogenous FOXS1 and miR-125a-5p levels were inversely correlated. miR-125a-5p can bind the 3′-UTR of FOXS1 and repress FOXS1 expression at the transcriptional level, consistent with the finding that miR-125a-5p mimics were able to inhibit FoxN1 3′UTR luciferase activity and suppress FoxN1 expression^39^. These results reveal that FOXS1 is also a target gene of miR-125a-5p and that its high expression might be due to the loss of miR-125a-5p, providing novel insight into miRNA regulation of human gene expression.

In summary, we first systematically identified FOXS1 as a novel oncogene and pinpointed the related regulatory network involved in FOXS1 in gastric cancer cells, providing new insight into understanding the mechanism underlying gastric cancer development. The findings of this study may potentially lead to the development of meaningful prognostic biomarkers for gastric cancer, and specific intervention strategies targeting FOXS1 might provide new therapeutic targets for gastric cancer patients.

## Materials and Methods

### Ethical approval and informed consent

All experimental procedures were approved by the first affiliated hospital of Chongqing Medical University and Chongqing Medical University. Written informed consent was obtained for all patient samples. The gastric tumor tissues and adjacent tissues were collected from 35 patients with gastric cancer at first affiliated hospital of Chongqing Medical University (during 2016 and 2017). The tissue microarray chips of gastric cancer (ST8014) were bought from Alenabio Biological Technology Co., Ltd. The study protocol was approved by the ethics committees at Chongqing medical university. Gastric cancer was diagnosed based on H&E staining and immune phenotype.

### Cell line and culture

The human gastric cancer cell lines (BGC823, MKN28, AGS, HGC-27 and SGC7901) were purchased from the Type Culture Collection of the Chinese Academy of Sciences (Shanghai, China). GES-1 was purchased from Obio Technology (Shanghai) Corp., Ltd. All the cells except AGS cultured in F12K medium were cultured in RPMI 1640 medium supplemented with 10% of fetal bovine serum, 100 U/ml of penicillin and 100 mg/ml of streptomycin. The cells were grown at 37 °C in a humidified incubator with 5% CO2. I confirm that the methods were performed in accordance with the indicating guidelines and regulations.

### Immunohistochemistry (IHC)

The human gastric cancer, adjacent tissue and tissues from heterologous tumor in nude mice were collected, fixed in 4% paraformaldehyde, embedded in paraffin, and sectioned (3μm). These paraffin sections were dewaxed, rehydrated and incubated in 3% H2O2 for 10 min to block endogenous peroxidase. Then the sections were autoclaved in 10 mM sodium citrate buffer (pH 6.0) for 15 min. Normal goat serum (10%) was used to block non-specific staining and then the tissue sections were exposed to indicated antibodies. For tissue microarray chips of gastric cancer versus gastric normal tissue, the target molecules were performed by using FOXS1 antibody. All of the staining were observed by at least 2 independent investigators blinded to the histopathologic features and patient data of the samples. I confirm that the methods were performed in accordance with the indicating guidelines and regulations.

### RNA extraction, reverse transcription, and Real-time PCR (RT-PCR)

Total RNA was extracted from tissues or cells using the Eastep ® Super Total RNA Extraction Kit (Promega Biotechnology, China), and the reverse transcription was performed using the PrimeScript™ RT Master Mix according to the manufacturer’s instructions (Takara Biotechnology, China). RT-PCR was performed by using the SYBR Green PCR Kit (Bio-Rad, China) in triplicate in three independent experiments by the 2−ΔΔCt method. The relative expression of miR-125a-5p or miR-328-3p were normalized to U6 and detected by Hairpin-it^TM^ miRNAs RT-PCR Quantitation Kit (Applied by Genepharma, Shang hai). RT-PCR primers sequences were showed as the Supplementary Table S1. I confirm that the methods were performed in accordance with the indicating guidelines and regulations.

### Western Blot (WB)

Western blot analysis was performed as previously described^45^. The FOXS1 primary antibody were purchased from SIGMA (HPA042475). E-cadherin (3195), N-cadherin (13116), β-catenin (8480), Vimentin (5741) and anti-rabbit IgG, HRP-linked antibody (7074) were purchased from CST. I confirm that the methods were performed in accordance with the indicating guidelines and regulations.

### Immunofluorescence (IF)

Cells were plated on 8-lm-thick chip, fixed in 4% ice-cold paraformaldehyde, and permeabilized using 0.5% Triton X-100/PBS for 20 min at room temperature. The cells were blocked with 10% albumin from bovine serum for 1 h and then incubated with anti-bodies against E-cadherin and N-cadherin (1:100, CST) overnight at 4 °C. After three washes, cells were incubated with Alexa Fluor 488-conjugated goat anti-rabbit secondary antibody (1: 1000; Invitrogen). The nuclei were counterstained with DAPI (1:100, Invitrogen), and cells were visualized with a laser scanning confocal microscope (Leica Microsystems, Wetzlar, Germany). I confirm that the methods were performed in accordance with the indicating guidelines and regulations.

### Cell proliferation assay

Cells were plated (3000 per well) in a 96-well plate. The absorbs value was measured at 490 nm by using CellTiter 96®AQ_ueous_ One Solution Cell Proliferation Assay kits (Promega, G3582) at indicating times. I confirm that the methods were performed in accordance with the indicating guidelines and regulations.

### Wound healing assay

The cells were seeded in 6-well plates and cultured to 100% confluence. Monolayer cells were washed with PBS, scraped with a plastic 200-ul pipette tip and then incubated with fresh 1640 medium without serum. The wounded areas were photographed by phase contrast microscopy at 24 h. The assay was done in triplicate at least three times. I confirm that the methods were performed in accordance with the indicating guidelines and regulations.

### SiRNA and transfection

The sequences of the siRNAs used to suppress FOXS1 expression and microRNA sequences were showed in Supplementary Table S2. Lipofectamine® RNAiMAX Reagent (Invitrogen) was used for transfection of miRNA or siRNA according to the manufacturer’s instructions. Lent virus5-GFP-FOXS1 was used to infect GES-1 or SGC7901 cells. After infection 24 h, cells were cultured in complete media containing puromycin (6 μg/ml) to generate a stable overexpressed cell line. I confirm that the methods were performed in accordance with the indicating guidelines and regulations.

### Dual- luciferase reporter assay

Transfection and luciferase reporter assay were performed as previously described^46^. The full-length and the different fragment sequences were synthesized and then cloned into the pGL3-basic vector. Nucleotide sequences of all the cloned DNA fragments were confirmed by direct DNA sequencing. I confirm that the methods were performed in accordance with the indicating guidelines and regulations.

### *In vivo* tumor angiogenesis assays

The animal study protocol was approved by the Animal Experimentation Ethics Committee of Chongqing Medical University. Six specific pathogen-free (SPF) BALB/c nude mice (4–6 week old) were obtained from the Institute of Medical Laboratory Animals, Chinese Academy of Medical Sciences. The mice were kept at 55 ± 5% humidity and 22–25 °C in Laboratory Animal Center of Chongqing medical university, fed with sterile water and food, and adaptively fed for 1 week before any experiment. SGC7901 cells (2 × 10^6^) infected with LV5-NC or LV5-FOXS1 virus were injected in the femoral area of the mice (n = 3/group). The tumor was measured with calipers and the volume was calculated using the formula: (π/6) × 3, where x = the largest diameter. Three weeks after tumor inoculation, the mice were sacrificed and the tumors were extracted to determine tumor weight. Data are presented as the mean ± SD. At the end, mice were sacrificed, the tumors were collected, fixed in 4% formaldehyde, sectioned for IHC staining, and observed under a microscope (Olympus, Tokyo, Japan). I confirm that the methods were performed in accordance with the indicating guidelines and regulations.

### Statistical analysis

All *in vitro* experiments were repeated three times or more, and data are presented as mean + SD. The student t test assumed two-tailed distributions to calculate statistical significance between groups. Survival curves were generated using the Kaplan–Meier method and compared using the log-rank tests. For analysis of correlation between FOXS1 levels and clinical features, Pearson’s chi-square tests were used. The independent prognostic factors were identified by the Cox proportional hazards regression model. ROC curve was generated with SPSS software. Differences were analyzed by GraphPad Prism 5. *P*-value < 0.05 was marked as statistically significant. *P*-value < 0.01 was indicated as highly statistically significant. *P*-value < 0.001 was indicated as extremely statistically significant difference. I confirm that the methods were performed in accordance with the indicating guidelines and regulations.

## Supplementary information

Supplementary Figures
