# Supplementary material for: FOXS1 is regulated by GLI1 and miR-125a-5p and promotes cell proliferation and EMT in gastric cancer
Source: Sci Rep. 2019 Mar 27;9:5281. doi: 10.1038/s41598-019-41717-w (PMC6437149; doi:10.1038/s41598-019-41717-w)
Supplement: Supplementary file 1 — Supplementary Figures [file 41598_2019_41717_MOESM1_ESM.pdf]

Supplementary Information for

FOXS1 is regulated by GLI1 and miR-125a-5p and promotes cell proliferation and EMT in gastric cancer

Sen Wang 1,2, Longke Ran 2,3#, Wanfeng Zhang 2,3#, Xue Leng 1,2#, Kexin Wang 4#, Geli Liu 1,2, Jing Song 2,3, Yujing Wang 1,2, Xianqin Zhang 1,2, Yitao Wang 1,2, Lian Zhang 1,2, Yan Ma 5, Kun Liu 1,2, Haiyu Li 6, Wei Zhang 7, Guijun Qin 8, Fangzhou Song 1,2*.

1 Molecular Medicine and Cancer Research Center, Chongqing Medical University, Chongqing 400016, China.
2 Department of Biochemistry and Molecular Biology, Chongqing Medical University, Chongqing 400016, China.
3 Department of Bioinformatics, Chongqing Medical University, Chongqing 400010, China
4 Department of Radiology, Affiliated Hospital of Southwest Medical University, Sichuan Province 646000, China
5 Information technology office of Chongqing medical university, Chongqing 400016,China
6 Chongqing public health medical center, Chongqing Medical University, Chongqing 400016, China.
7 Department of Gastrointestinal Surgery, The First Affiliated Hospital of Chongqing Medical University, Chongqing, China.
8 Department of Endocrinology of the Frist Affiliated Hospital of Zhengzhou University, Zhengzhou, Henan, 450052, PR China.
# These authors contributed equally to this work.
*Correspondence to: Prof. Fangzhou Song, Department of Biochemistry and Molecular Biology, and Molecular Medicine and Cancer Research Center, Chongqing Medical University, Chongqing 400016, P. R. China. Email: fzsongcq@163.com. Telephone number: +86-13509419106

Figure S1

The expression of FOXS1 in tissue chip of ST8014

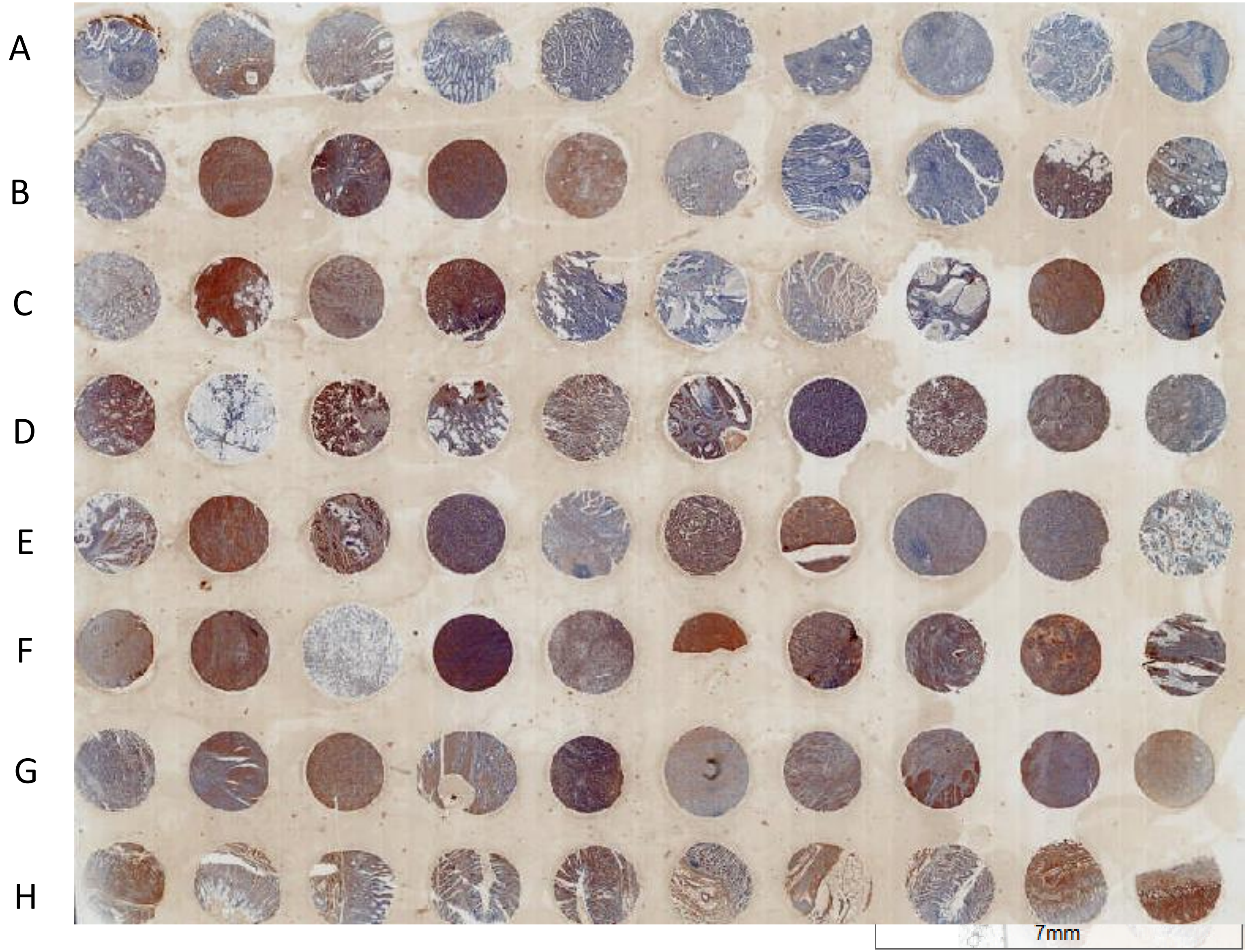

Figure S1. FOXS1 expression in tissue chip of ST8014 was detected by immunohistochemical staining. Detailed medical records and information of each cases in tissue chip can be checked at this website (http://www.alenabio.com/tissue-array/Stomach/ST8014).

Figure S2 Hematoxylin-eosin (HE) staining in tissue chip of ST8014

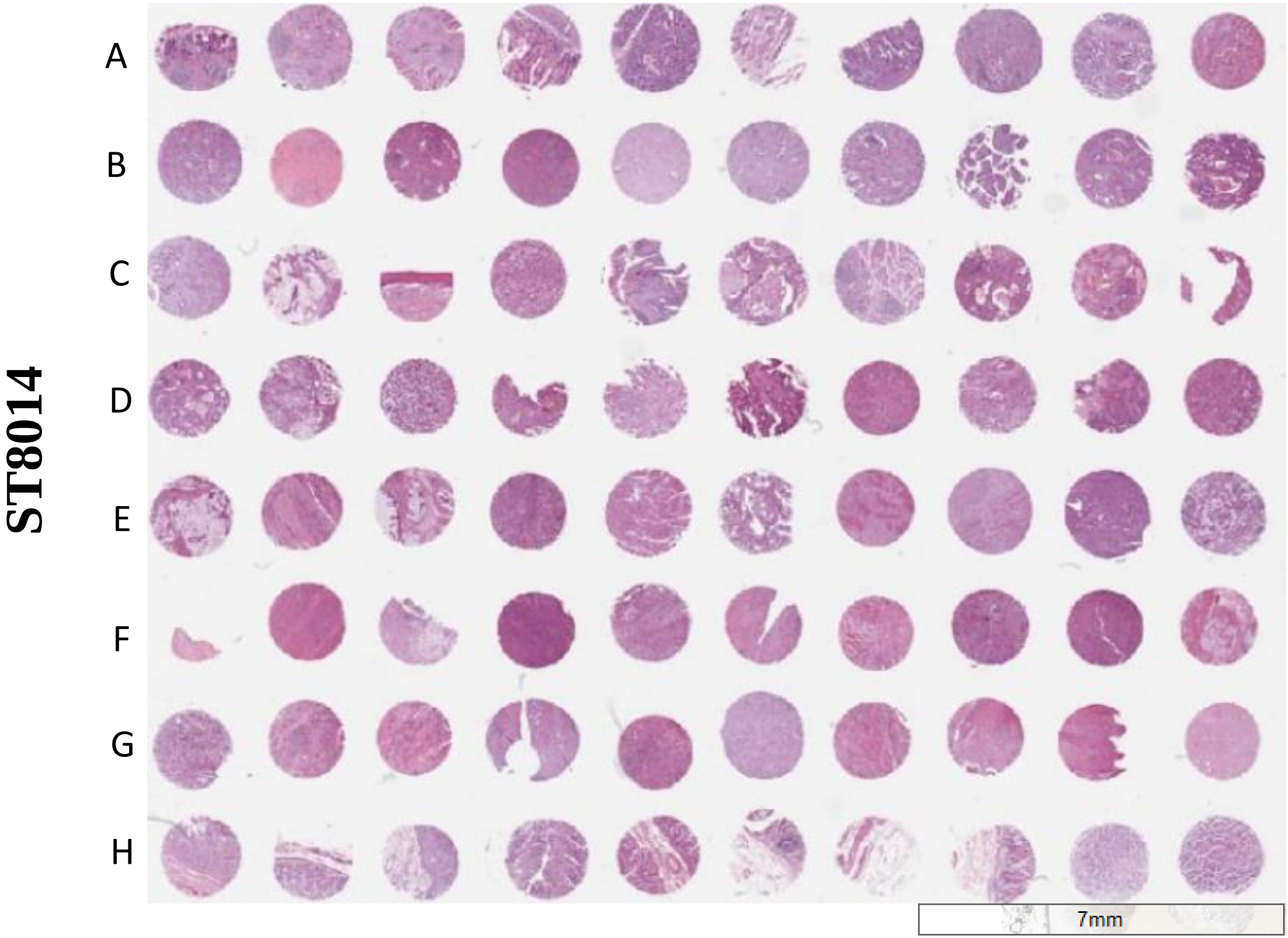

Figure S2. Hematoxylin-eosin (HE) staining in tissue chip of ST8014 were detected by immunohistochemistry. The enlarged figure of each case can be seen at this website (http://www.alenabio.com/tissue-array/Stomach/ST8014).

Figure S3

The distribution of gastric cancer and gastric normal tissue in ST8014 tissue chip

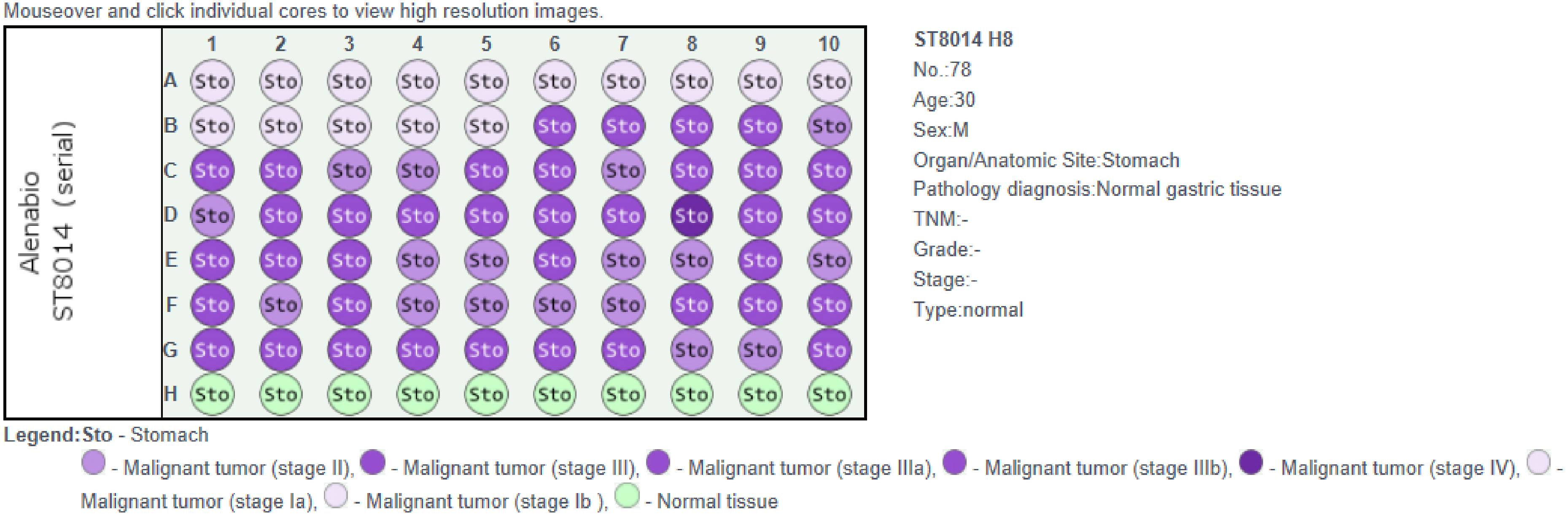

Figure S3. The distribution of gastric cancer and gastric normal tissue in ST8014 tissue chip. Detailed medical records and information of each cases in tissue chip can be checked at this website (http://www.alenabio.com/tissue-array/Stomach/ST8014).

Figure S4

Effects of gene overexpression on cell proliferation and cloning in SGC7901 cells

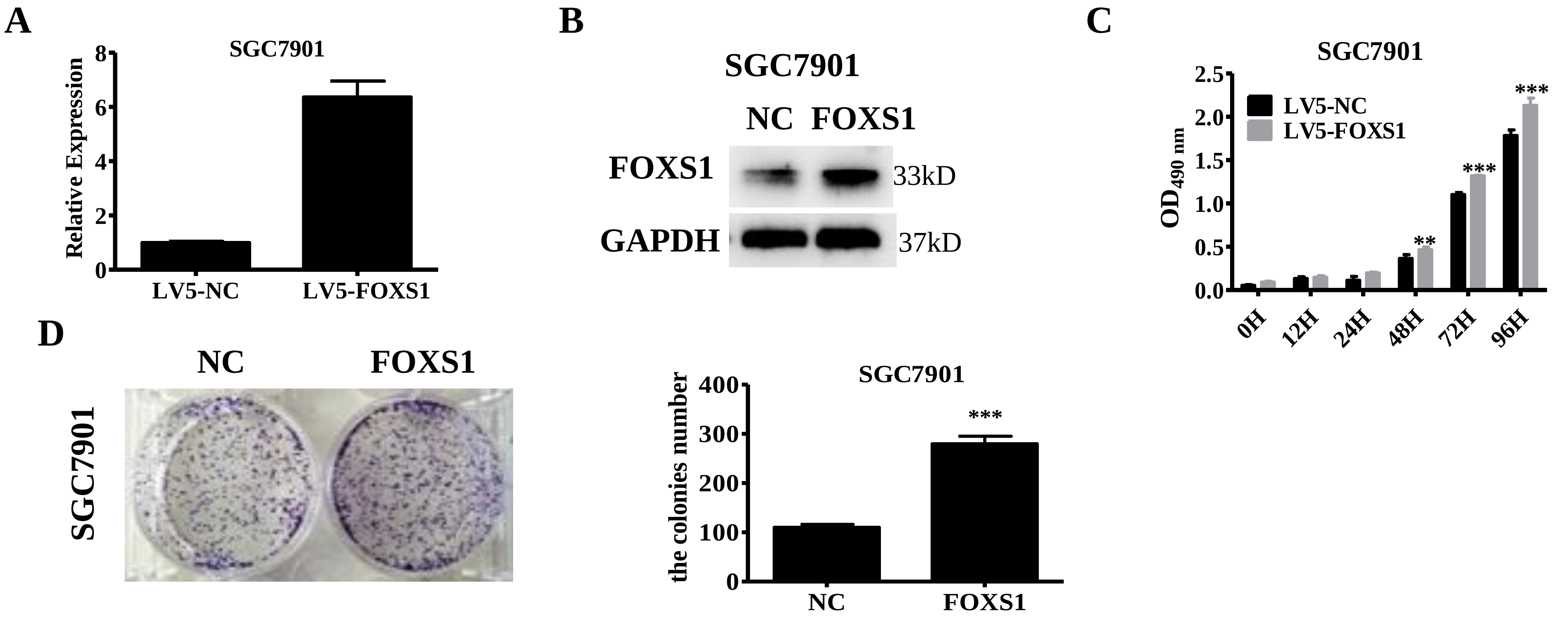

Figure S4. (A and B) Overexpression effect of FOXS1 at mRNA levels was confirmed by RT-PCR and at protein levels was confirmed by WB. (C) Effect of FOXS1 overexpression on cell viability were detected by Cell Proliferation Assay kits. (D) The effect of FOXS1 overexpression on cell colony formation was analyzed by Crystal violet staining, and quantitative analysis of cell colony numbers was showed in the right panel.

Figure S5

The expression of c-Myc and Cyclin D1 detected by RT-PCR

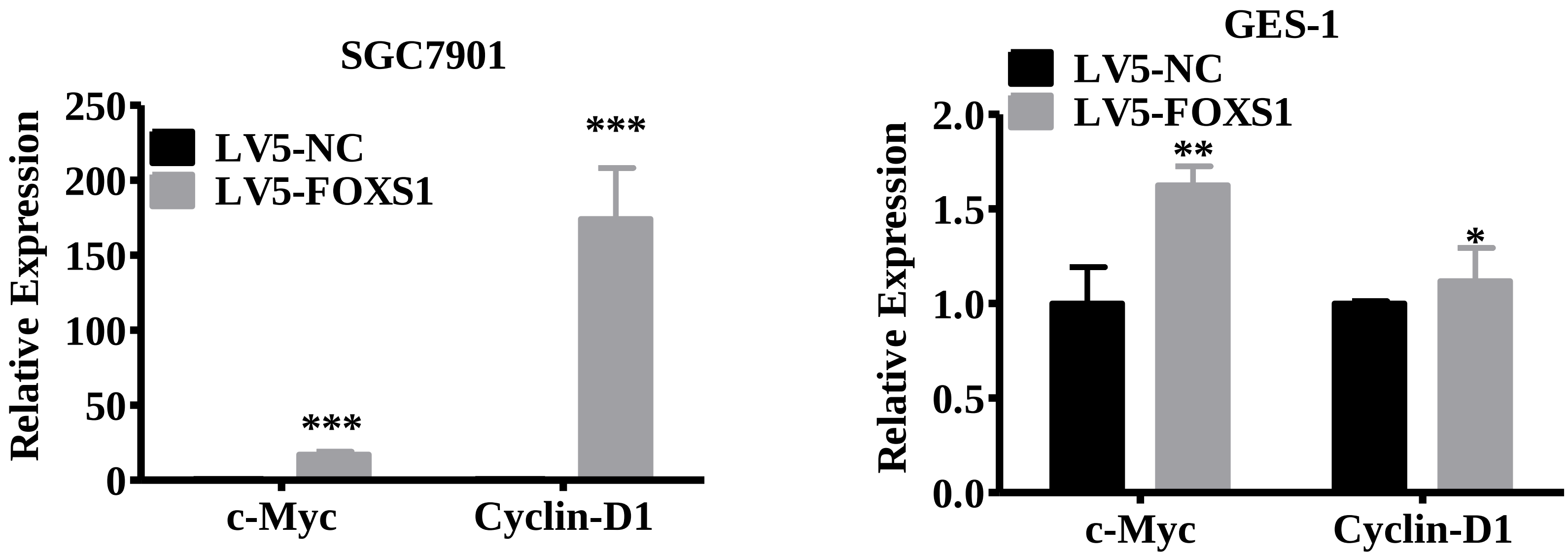

Figure S5. The expression of c-Myc and Cyclin D1 detected by RT-PCR in SGC7901 or GES-1 cells which were respectively infected with LV5-FOXS1 or controls LV5-NC.

Figure S6

The expression of GLI1 in gastric cancer cells

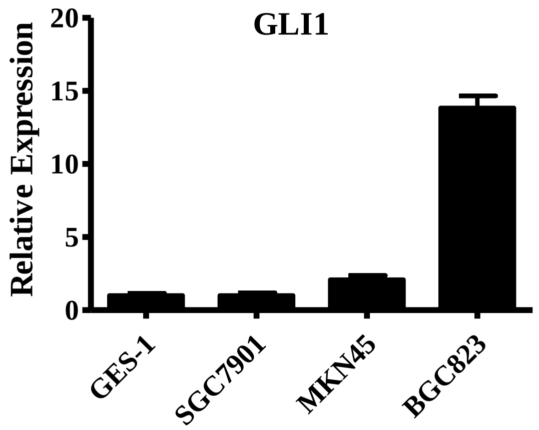

Figure S6. GLI1 expression in four indicating kinds of gastric cancer cells were determined by RT-PCR, GAPDH was internal control.

Figure S7

The effect of GLI1 on the FOXS1 promoter regions in SGC7901 cells

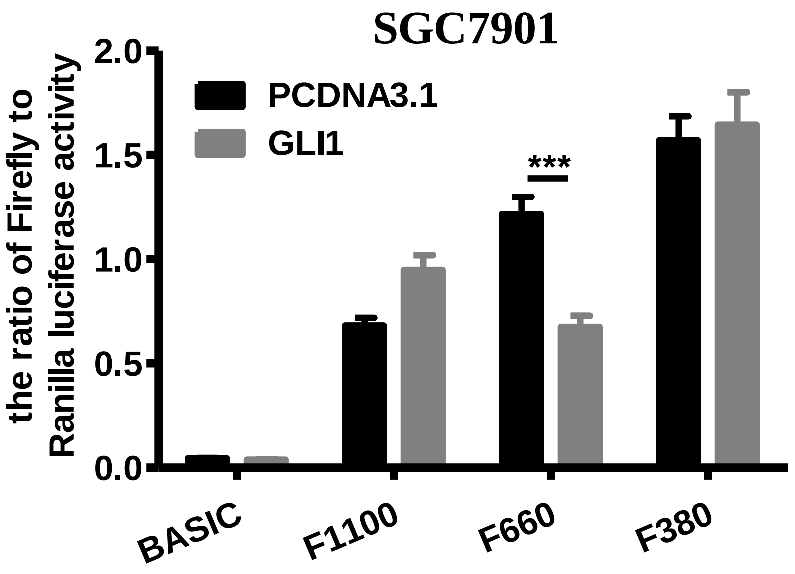

Figure S7. The effect of transcription factors GLI1 on luciferase activity of FOXS1 promoters F1100，F660 and F380 detected by luciferase reporter assays.

Figure S8

The expression of miRNA in gastric cancer cells

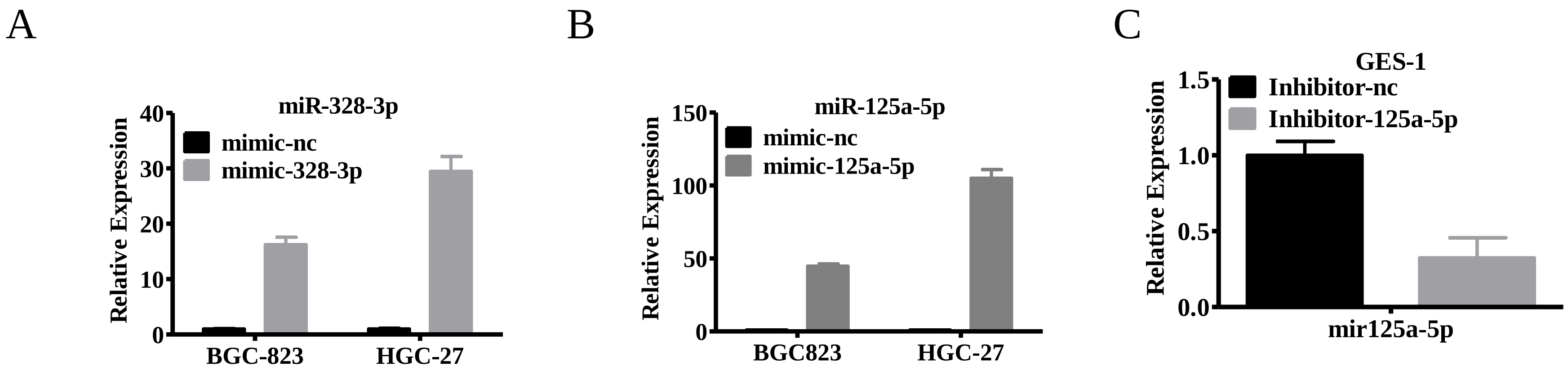

Figure S8. (A) RT-PCR results of the expression of miR-328-3p in indicated cells transfected with mimics of miR-328-3p or nc. (B) RT-PCR results of the expression of miR-125a-5p in indicated cells transfected with mimics of miR-125a-5p or nc. (C) RT-PCR results of the expression of miR-125a-5p in GES-1 cells transfected with inhibitors of miR-125a-5p or nc.

Figure S9

The relative luciferase activity of the FOXS1 promoter regions

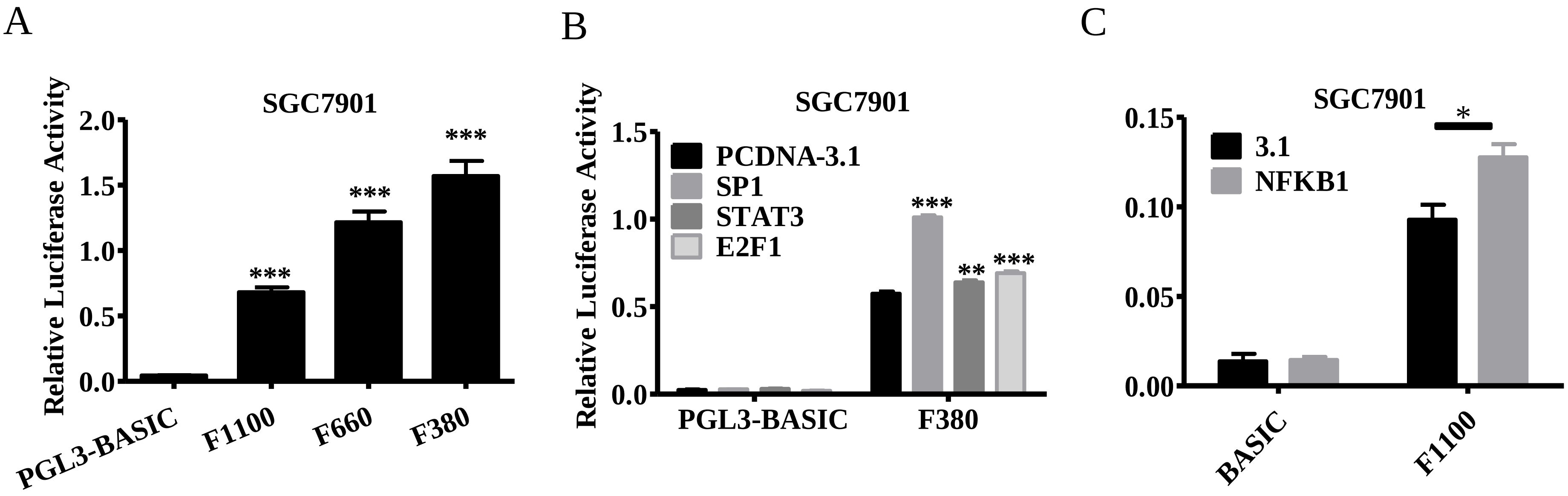

Figure S9. (A) The relative luciferase activity of the luciferase reporter vectors with FOXS1 promoter regions of F1100, F660, F380 and control luciferase reporter vectors in SGC7901detected by dual luciferase reporter assays. (B) The effect of transcription factors STAT3, E2F1 and SP1 on luciferase activity of FOXS1 promoters F380 detected by luciferase reporter assays. (C) The effect of transcription factors NFKB1 on luciferase activity of FOXS1 promoters F1100 detected by luciferase reporter assays.

Table S1. List of qRT-PCR Primer Sequences

Gene name	Primer sequences
GAPDH	(F) 5’-AGAAGGCTGGGGCTCATTTG-'3
	(R) 5’-AGGGGCCATCCACAGTCTTC-'3
FOXS1	(F) 5’-GCTACATCATGGGCCGATTC-'3
	(R) 5’- CAAACATGTCGTGGCAGTCA-'3
c-Myc	(F) 5’- AACACACAACGTCTTGGAGC-'3
	(R) 5’- GCACAAGAGTTCCGTAGCTG-'3
Cyclin D1	(F) 5’- GCATGTTCGTGGCCTCTAAG-'3
	(R) 5’- CGTGTTTGCGGATGATCTGT-'3
NF-KB-P65	(F) 5’-TGGCCCCTATGTGGAGATCA-'3
	(R) 5`-AGGGGTTGTTGTTGGTCTGG-'3
GLI1	(F) 5`-TCCTACCAGAGTCCCAAGTT-'3
	(R) 5`-CCCTATGTGAAGCCCTATTT-'3

Table S2. List of Small Interference RNA Sequences

**Gene or miRNA name**	**siRNA sense sequences**
siFOXS1-212	5'-GCUACAUCAUGGGCCGAUUTT-'3
siFOXS1-1040	5'- GGACGCCAGGAAUGUUCUUTT-'3
siFOXS1-137	5'- ACAGCUACAUCGCCCUUAUTT-'3
siFOXS1-275	5'- GCCACAACCUGUCACUCAATT-'3
siFOXS1-641	5'-CCAUGGAGCCCAAAGAGAUTT-'3
siGLI1-2758	5'-GGCUCAGCUUGUGUGUAAUTT-'3
siNC	5'-UUCUCCGAACGUGUCACGUUU-'3
MicroRNA inhibitors-NC	5'-CAGUACUUUUGUGUAGUACAA-'3
MicroRNA inhibitors of hsa-miR-125a-5p	5'-UCACAGGUUAAAGGGUCUCAGGGA-'3
MicroRNA mimics-NC	5'-UUGUACUACACAAAAGUACUG-'3
MicroRNA mimics of has-miR-125a-5p	5'-UCCCUGAGACCCUUUAACCUGUGA-'3
MicroRNA mimics of has-miR-328-3p	5'-CUGGCCCUCUCUGCCCUUCCGU-'3
